# Ca^2+^/Calmodulin-Dependent Protein Kinase II Inhibits Hepatitis B Virus Replication from cccDNA via AMPK Activation and AKT/mTOR Suppression

**DOI:** 10.3390/microorganisms10030498

**Published:** 2022-02-23

**Authors:** Jumi Kim, Hyeonjoong Kwon, Fadia Kalsoom, Muhammad Azhar Sajjad, Hyun Woong Lee, Jin Hong Lim, Jaesung Jung, Yong-Joon Chwae, Sun Park, Ho-Joon Shin, Kyongmin Kim

**Affiliations:** 1 Department of Microbiology, Ajou University School of Medicine, Suwon 16499, Korea; jumi9143@gmail.com (J.K.); hyunjeing@naver.com (H.K.); fadiakalsoom@gmail.com (F.K.); azharsajjad71@gmail.com (M.A.S.); jjsbio118@gmail.com (J.J.); soiloie0603@hanmail.net (Y.-J.C.); sinsun@ajou.ac.kr (S.P.); hjshin@ajou.ac.kr (H.-J.S.); 2 Department of Biomedical Science, Graduate School of Ajou University, Suwon 16499, Korea; 3 Department of Internal Medicine, Gangnam Severance Hospital, Yonsei University College of Medicine, Seoul 06273, Korea; lhwdoc@yuhs.ac; 4 Department of General Surgery, Gangnam Severance Hospital, Yonsei University College of Medicine, Seoul 06273, Korea; doctorjin@yuhs.ac; 5 GC Pharma, Ihyeon-ro 30-beon-gil, Giheung-gu, Yongin-si 16924, Korea

**Keywords:** hepatitis B virus, HBV replication, hepatocellular carcinoma, CaMKII, AMPK, AKT/mTOR signaling pathway, HBx

## Abstract

Ca^2+^/calmodulin-dependent protein kinase II (CaMKII), which is involved in the calcium signaling pathway, is an important regulator of cancer cell proliferation, motility, growth, and metastasis. The effects of CaMKII on hepatitis B virus (HBV) replication have never been evaluated. Here, we found that phosphorylated, active CaMKII is reduced during HBV replication. Similar to other members of the AMPK/AKT/mTOR signaling pathway associated with HBV replication, CaMKII, which is associated with this pathway, was found to be a novel regulator of HBV replication. Overexpression of CaMKII reduced the expression of covalently closed circular DNA (cccDNA), HBV RNAs, and replicative intermediate (RI) DNAs while activating AMPK and inhibiting the AKT/mTOR signaling pathway. Findings in HBx-deficient mutant-transfected HepG2 cells showed that the CaMKII-mediated AMPK/AKT/mTOR signaling pathway was independent of HBx. Moreover, AMPK overexpression reduced HBV cccDNA, RNAs, and RI DNAs through CaMKII activation. Although AMPK acts downstream of CaMKII, AMPK overexpression altered CaMKII phosphorylation, suggesting that CaMKII and AMPK form a positive feedback loop. These results demonstrate that HBV replication suppresses CaMKII activity, and that CaMKII upregulation suppresses HBV replication from cccDNA via AMPK and the AKT/mTOR signaling pathway. Thus, activation or overexpression of CaMKII may be a new therapeutic target against HBV infection.

## 1. Introduction

Hepatitis B virus (HBV), a prototype virus of the *Hepadnaviridae* family, contains a small, partially double-stranded, relaxed 3.2 kb circular (RC) DNA [1]. Although an effective vaccine is available, HBV infection remains a public health concern. Chronic hepatitis B (CHB) infection, which has been estimated to affect 257 million individuals worldwide (WHO), is closely associated with cirrhosis and hepatocellular carcinoma (HCC) [2]. Upon HBV infection of a cell, its RC DNA is translocated to the nucleus and converted into a covalently closed circular DNA (cccDNA), which serves as the template for transcription of viral RNAs, including 3.5 kb pregenomic RNA (pgRNA) and subgenomic RNAs (sgRNAs) [3]. Translation of the 2.4 kb, 2.1 kb, and 0.7 kb sgRNAs produces large, middle, and small HBV surface (HBs) proteins and HBV X (HBx) proteins, respectively [4]. Viral pgRNA is translated in the cytoplasm to produce HBV core (HBc) and polymerase proteins [5].

HBV has been shown to induce metabolic reprogramming to provide energy for HBV replication [6,7,8]. HBV infection increases glucose uptake, glycolytic metabolism, and lipid accumulation, which drive hepatic tissue damage resulting in the development of cirrhosis and HCC [9,10,11]. Although many metabolic proteins are involved in HBV replication [6,7,8,9,10,11], the underlying signaling pathway associated with metabolism during HBV replication has not yet been clearly determined. Evaluation of the metabolic signaling pathways associated with HBV replication showed that the AMPK activator metformin, which is used to treat patients with type 2 diabetes, suppressed the syntheses of HBe and HBs antigens and HBV replicative intermediate (RI) DNAs [12]. In addition, the AMPK activator 5-aminoimidazole-4-carboxamide ribonucleotide (AICAR) suppressed HBV replication through autophagosome degradation [13]. HBV replication in hepatocytes has been reported to stimulate the phosphorylation of AKT and mTOR to control glucose uptake [14,15]. Although AMPK [12,13,15], AKT, and mTOR [14,15] were shown to be involved in HBV replication, the genes acting upstream of the AMPK/AKT/mTOR signaling pathway have not yet been investigated. 

The multimeric serine/threonine protein kinase, Ca^2+^/calmodulin-dependent protein kinase II (CaMKII) is a critical controller that regulates learning, memory, and cognition [16]. CaMKII has four isoforms, CaMKII α, β, γ, and δ, with different functions [17]. CaMKII α is abundant in the brain but is also expressed in the liver, heart, kidney, lung, and smooth muscle [18,19]. CaMKII is composed of catalytic, autoregulatory, and association domains [20,21]. The catalytic domain contains binding sites for ATP and substrate protein, transferring a phosphate moiety of ATP to residues of the substrate [22]. The autoregulatory domain, as a pseudosubstrate, interacts with the catalytic domain and prevents the binding of substrate proteins [23]. The association domain is responsible for the assembly of subunits to form the holoenzyme [24]. Formation of the 12meric CaMKII holoenzyme is mediated by the binding of Ca^2+^/calmodulin to the autoregulatory domain, allowing ATP and substrate proteins to interact with the catalytic domain [25]. CaMKII autophosphorylation at T286 triggers permanent activation [26,27]. The activation of CaMKII has been shown to inhibit the metastasis of HCC [28,29]. 

The present study evaluated the effects of CaMKII on HBV replication, finding that HBV replication decreased the amount of active, phosphorylated CaMKII in these cells. Conversely, overexpression of CaMKII inhibited HBV replication in an HBx-independent manner while activating AMPK and inhibiting AKT/mTOR. This study also showed that CaMKII and AMPK form a feedback loop, affecting the AKT/mTOR signaling pathway during HBV replication. Moreover, overexpression of either CaMKII or AMPK reduced HBV cccDNA levels, suppressing the syntheses of HBV RNAs and RI DNAs. Taken together, these findings suggested that the activation of either CaMKII or AMPK might be a new therapeutic target for the treatment of patients with CHB and HCC.

## 2. Materials and Methods 

### 2.1. DNA Constructs

Wild type (WT) and HBx-deficient mutant constructs of replication-competent 1.3 mer HBV subtype ayw were kindly provided by Dr. Wang-Shick Ryu (Yonsei Univ, South Korea). pCDH-hNTCP-C9 generated from the human NTCP-C9 (hNTCP-C9) construct in pcDNA6.1, kindly provided by W. Li [30], has been described [31]. The codon optimized CaMKII α gene was synthesized and inserted into an *Eco*R I-linearized pBHA plasmid, resulting in the plasmid pBHA-CaMKII α. To generate pCMV10-HA-CaMKII α, PCR-amplified CaMKII α DNA (Table S1) from pBHA-CaMKII α was subcloned into the *Eco*R I-linearized vector pCMV10-HA. To generate pCMV10-Myc-AMPK α1, PCR-amplified AMPK α1 DNA from pHA-AMPK α1 (provided by Dr. Joohun Ha) [32] (Table S1) was subcloned into the *Msc* I- and *Eco*R I-linearized vector pCMV10-Myc. A lentiviral expression vector encoding the HA-CaMKII α gene was generated by inserting *Xba* I- and *Bam*H I-digested HA-CaMKII α DNA into the linearized pCDH-CMV-MCS-EF1-Puro (System Biosciences; CD510B-1) vector, and a lentiviral encoding the Myc-AMPK α1 gene was generated by inserting *Xba* I- and *Eco*R I-digested Myc-AMPK α1 DNA into linearized pCDH-CMV-MCS-EF1-Puro. The luciferase reporter pGL3-EnhII/Cp, pGL3-EnhI/Xp, pGL3-preS1p, and pGL3-preS2p constructs have been described previously [33].

### 2.2. Cell Culture

Huh7, HepG2, HepG2-hNTCP-C9, HepG2.2.15, HepAD38, and HEK293T were maintained in Dulbecco’s modified Eagle’s medium (DMEM; Gibco) supplemented with 10% (*v*/*v*) heat inactivated fetal bovine serum (FBS; Gibco) and 1% (*v*/*v*) penicillin-streptomycin (Gibco) under a humidified atmosphere at 37 °C containing 5% CO_2_. Cells were passaged as described previously [31]. HepG2.2.15 cells were maintained as described above, except that 1 mg/mL G418 was added for selection [34,35]. HepAD38 cells were also maintained as above, except that 2 μg/mL tetracycline (TC) was added and subsequently removed to induce HBV transcription [36,37]. 

### 2.3. Transfection

Huh7 cells (1 × 10^6^) were plated in 6 cm dishes and transfected 24 h after seeding with 6 μg of 1.3 mer HBV WT (ayw) using 18 μg polyethylenimine (PEI; Polysciences; Warrington, Pennsylvania, USA; 23966-2) in 200 μL Opti-MEM (Thermo Fisher Scientific; Waltham, MA, USA; 31985062). HepG2 cells (2 × 10^6^) in 6 cm dishes were transfected 24 h after seeding with 8 μg of 1.3 mer HBV WT (ayw) using 40 μg PEI in 200 μL Opti-MEM. For co-transfection into Huh7 cells, 3 μg of 1.3 mer HBV WT (ayw) plus 3 μg HA-CaMKII α or Myc-AMPK α1 were mixed with 18 μg PEI and 200 μL Opti-MEM and transfected into Huh7 cells (1 × 10^6^) plated in 6 cm dishes 24 h after cell-seeding. For co-transfection into HepG2 cells, 4 μg of 1.3 mer HBV WT (ayw) plus 4 μg HA-CaMKII α or Myc-AMPK α1 were mixed with 40 μg PEI in 200 μL Opti-MEM and transfected into HepG2 cells (2 × 10^6^) in 6 cm dishes 24 h after cell-seeding. To determine the effects of CaMKII α or AMPK α1 on 1.3 mer HBV X-null replication, 4 μg of 1.3 mer HBV WT (ayw) or HBx-deficient mutant constructs were co-transfected into 2 × 10^6^ HepG2 cells with 4 μg of either HA-CaMKII α or Myc-AMPK α1. Culture medium was replaced by fresh medium 24 h after transfection, and the cells were lysed 72 h after transfection.

### 2.4. Establishment of Stable Cells

HepG2-hNTCP-C9 stable cells were generated as previously reported [31,33,38]. HepG2-hNTCP-C9-HA-CaMKII α and -Myc-AMPK α1 stable cells were generated using a lentiviral expression system. Briefly, 1 × 10^6^ HEK293T cells in 10 cm dishes were co-transfected with 1 μg pVSV-G and 3 μg pGAG-pol plus 4 μg of either pCDH empty, pCDH-HA-CaMKII α, or pCDH-Myc-AMPK α1 using 16 μg PEI in 500 μL Opti-MEM. Twenty-four h later, the culture medium was replaced with fresh medium. Supernatants containing pseudoviral particles with HA-CaMKII α or Myc-AMPK α1 transcript were harvested 72 h after transfection. Two ml of supernatant containing pseudovirus particles plus 2 mL of culture medium containing 20 μg/mL of polybrene (Sigma Aldrich; St. Louis, MO, USA; H9268) were added to 2 × 10^6^ HepG2-hNTCP-C9 cells in 6 cm dishes. Twenty-four h later, the culture medium was replaced, and 48 h after transduction, the cells were selected with 10 μg/mL puromycin (Sigma Aldrich; St. Louis, MO, USA; P8833).

### 2.5. SDS-PAGE and Western Blotting

Cells were lysed in 0.2% NP-40 (IGEPAL)-TNE (10 mM Tris-HCl [pH 8.0], 50 mM NaCl, 1 mM EDTA) buffer with 1 × protease inhibitor cocktail (Calbiochem; San Diego, CA, USA; 535142) and 1 × phosphatase inhibitor cocktail (Sigma Aldrich; St. Louis, MO, USA; P0044). Tumor and non-tumor liver biopsy specimens from patients with HBV-associated HCC were obtained after surgery from Gangnam Severance Hospital, Yonsei University College of Medicine. All patients provided written informed consent, and the study protocol was approved by the institutional review board of Yonsei University College of Medicine (3-2019-0031). Paired tumor and non-tumor liver tissue specimens were chopped into small pieces and lysed in ice-cold M-PER mammalian protein extraction reagent (Thermo Fisher Scientific; Waltham, MA, USA; 78501) with 1 × protease and phosphatase inhibitor cocktail (Thermo Fisher Scientific; Waltham, MA, USA; 78440). On the ice, the samples were homogenized with a Dounce homogenizer and centrifuged at 13,400× *g* for 15 min. The supernatants were collected, and their protein concentrations were determined using the Bradford assay [39]. Proteins were separated by sodium dodecyl sulfate-12% polyacrylamide gel electrophoresis (SDS-PAGE) and transferred to PVDF membranes. The membranes were blocked with 4% skim milk, incubated with primary antibodies (1:1000) (Table S2), washed, and incubated with horseradish peroxidase (HRP)-conjugated goat anti-rabbit IgG (1:5000; Thermo Fisher Scientific; Waltham, MA, USA; 31460) or goat anti-mouse IgG (1:5000; KPL; New Delhi, India 474-1802) secondary antibodies. The bands were visualized using ECL (GE Healthcare Life Sciences; Piscataway, NJ, USA; RPN2106) and their relative intensities were measured using ImageJ 1.50b software.

### 2.6. Core Particle Immunoblotting

Cell lysates in 0.2% NP-40-TNE buffer were separated by 1% native agarose gel electrophoresis (NAGE) and transferred to PVDF membranes. The membranes were blocked with 4% skim milk, incubated with rabbit polyclonal anti-HBc (1:1000) antibody [40], washed, and incubated with HRP-conjugated goat anti-rabbit IgG (1:5000). The bands were visualized using ECL and their relative intensities were measured using ImageJ 1.50b software.

### 2.7. Northern and Southern Blotting

Total RNAs were obtained using 1 mL Trizol (Ambion; Austin, Texas, USA; 15596018) according to the manufacturer’s instructions, and the dried RNA pellets were dissolved in nuclease free water (Ambion; Austin, Texas, USA; AM9932). Aliquots containing 20 μg total RNA were denatured at 65 °C for 10 min and then separated by 1% agarose gels (Invitrogen; Waltham, MA, USA; 16500100) with 18% formaldehyde (Sigma Aldrich; St. Louis, MO, USA; F8775) and 1 × morpholinopropanesulfonic acid (MOPS) buffer (200 mM MOPS, 10 mM EDTA, 50 mM sodium acetate [pH 7.0]). RNAs were transferred to nylon membranes (Roche; Basel, Switzerland; 11417240001) and hybridized with a random-primed ^32^P-labeled probe specific for full-length HBV sequences [41]. 

For Southern blotting, HBV DNAs from isolated core particles were separated by 1% NAGE, transferred to nylon membranes (Roche; Basel, Switzerland; 11417240001), and hybridized with a random-primed ^32^P-labeled probe specific for full-length HBV sequences, as described above [41]. The relative intensities of HBV pregenomic RNA (pgRNA), subgenomic RNAs (sgRNAs), and RI DNAs, including RC, double-stranded linear (DL), and single-stranded (SS) DNAs, were measured using ImageJ 1.50b software. 

### 2.8. HBV Preparation and Infection

HBV virions for infection were prepared from HepAD38 cells as described, with minor modifications [33,38,42]. Briefly, cells were maintained in DMEM supplemented with 10% FBS, 50 µM hydrocortisone hemisuccinate, 5 µg/mL insulin, and 1% penicillin-streptomycin. The culture supernatants were collected every second day from day 7 to 21 and precipitated with 6% polyethylene glycol (PEG; Affymetrix; Santa Clara, CA, USA; 25322-68-3) at 4 °C overnight. The virions in the PEG pellets were resuspended in phosphate-buffered saline (PBS) at 4 °C for 4 h and clarified by centrifugation at 3000× *g* at 4 °C for 10 min to remove insoluble materials [43]. The clarified PEG pellets were ultracentrifuged at 26,000× *g* on 20% sucrose cushion and resuspended in DMEM supplemented with 10% FBS, 50 µM hydrocortisone hemisuccinate, 5 µg/mL insulin, and 1% penicillin-streptomycin. HBV virion DNAs were quantitated by Southern blotting. 

For HBV infection, 3 × 10^6^ HepG2, HepG2-hNTCP-C9, and HepG2-hNTCP-C9-HA-CaMKII α, and/or -Myc-AMPK α1 cells in collagen-coated (Corning; Corning, NY, USA; 354249) 10 cm dishes were infected with 2 × 10^2^ genome equivalents (GEq) of HBV virions per cell in medium containing 4% PEG and 2.5% dimethyl sulfoxide (DMSO). After 24 h, the cells were washed with PBS and maintained in the same, fresh medium containing 2.5% DMSO [44]. The cells were harvested 7 days after infection.

### 2.9. cccDNA Extraction 

HBV cccDNA was extracted using the Hirt protein-free DNA extraction procedure, as described previously [45]. Briefly, 3 × 10^6^ HepG2, HepG2-hNTCP-C9, and HepG2-hNTCP-C9-HA-CaMKII α, or -Myc-AMPK α1 cells in collagen-coated 10 cm dishes were infected with HBV as described above. Seven days after infection, the cells were lysed with 0.6% SDS-TE buffer (10 mM Tris-HCl [pH 7.5], 10 mM EDTA) for 30 min at room temperature. The NaCl concentrations of the lysates were adjusted to 1 M with 5 M NaCl, and the lysates were incubated at 4 °C for 16 h, and centrifuged at 14,500× *g* for 30 min. The supernatants containing cccDNA were extracted twice with phenol and once with phenol-chloroform, followed by ethanol precipitation, with cccDNA quantified by Southern blotting. To further validate the authenticity of HBV cccDNA, the Hirt DNA sample was heated to 85 °C for 5 min. Among three forms of HBV DNA from the Hirt protein-free DNA extraction sample, including cccDNA (2.1 Kbp), DL DNA (3.2 Kbp), and protein-free RC DNA (above 3.2 Kbp), RC and DL DNAs were denatured but cccDNA was not. Electrophoretic mobility of cccDNA remains unchanged. Then, heat-treated DNA sample was further digested with *Eco*R I to linearize cccDNA to a genome-length double-stranded DNA (3.2 Kbp).

### 2.10. Drug Treatment

HepG2, HepG2.2.15, and HepAD38 cells (1 × 10^6^) were plated onto 6 cm plates and treated 24 h later with 2 mM or 4 mM 1, 1-dimethylbiguanide hydrochloride (metformin; AMPK activator; Sigma Aldrich; St. Louis, MO, USA; D150959) in distilled water or 1 mM or 2 mM 5-aminoimidazole-4-carboxamide-1-h-D-ribofuranoside (AICAR; AMPK activator; Toronto Research Chemicals, Toronto, ON, Canada; A611700) in distilled water. After treatment for 24 h, the cells were lysed with 0.2% NP-40-TNE buffer and analyzed as described above. 

In addition, 1 × 10^6^ Huh7 cells in 6 cm plates were seeded and (co-)transfected as described above. Twenty-four h after transfection, the cells were treated for 48 h with 10 μM KN93 (CaMKII inhibitor; Santa Cruz; Santa Cruz, CA, USA; sc2502199) in DMSO, 5 μM compound C (AMPK inhibitor; Calbiochem; San Diego, CA, USA; 171260) in DMSO, or 2 mM metformin in distilled water. The cytotoxic effects of these reagents were determined by MTT (3-[4,5-dimethylthiazol-2-yl]-2,5-diphenyltetrazolium bromide) assays, as described previously [46].

### 2.11. Luciferase Reporter Assay

HepG2 cells (1 × 10^6^) were seeded in 6-well plates and co-transfected with 2 μg pGL3-null, pGL3-EnhII/Cp, pGL3-EnhI/Xp, pGL3-preS1p, or pGL3-preS1p plus 2 μg of either HA-CaMKII α or Myc-AMPK α1. Forty-eight h after transfection, the cells were lysed with 1 × luciferase cell culture lysis reagent (Promega; Madison, WI, USA; E153A). Luciferase activities were analyzed using luciferin (Promega; Madison, WI, USA;) and a luminometer (Molecular Devices; BioTek; Winooski, VT, USA; EPOCH2NS). The results shown are the mean luciferase activities from three independent experiments. 

### 2.12. Quantitative Real-Time RT-PCR and PCR

To analyze mRNA levels of CaMKII and AMPK in HBV replicating cells, 1.3 mer HBV WT (ayw) was transiently transfected in HepG2 and Huh7 cells and total RNAs were isolated as described above. Total RNAs were adjusted to 1 μg using a spectrophotometer (Thermo Fisher Scientific; ND-ONEC-W) and was reverse transcribed to cDNA using SuperScript™ III Reverse Transcriptase (Invitrogen; Waltham, MA, USA; 18080093) according to the manufacturer’s instructions. Quantitative real-time PCR (Applied Biosystems by Thermo Fisher Scientific, QuantStudio 3 Real-Time PCR; A28131) was performed using CaMKII- or AMPK-specific primers (Table S1). The relative mRNA levels were quantified by normalization to actin (loading control).

HBV cccDNA in HBV-infected cell was analyzed by quantitative real-time PCR. Briefly, HBV was infected in HepG2-hNTCP-C9, and HepG2-hNTCP-C9-HA-CaMKII α, and/or -Myc-AMPK α1 cells and then HBV cccDNA was extracted as described above. The DNA amount was adjusted to 50 ng and quantitative real-time PCR was performed using cccDNA-specific primers (Table S1) as described above. The cccDNA levels were quantified by normalization to actin (loading control).

### 2.13. Statistical Analysis 

Data were reported as the means ± SD from at least three independent experiments. Mean values were compared using Student’s *t*-test for most experiments, and GraphPad Prism version 5 for luciferase reporter assay. *p*-values < 0.05 were considered statistically.

## 3. Results

### 3.1. Active, Phosphorylated CaMKII Is Decreased in HBV Replicating Cells 

**Figure 1:**
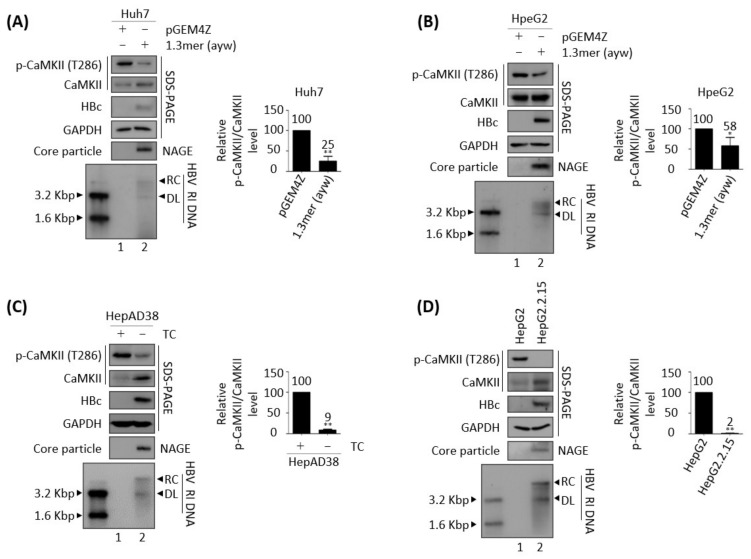
Association between HBV replication and reduced CaMKII phosphorylation. (**A**) Huh7 and (**B**) HepG2 cells were transfected with pGEM4Z (lane 1) or 1.3 mer HBV WT (ayw) (lane 2) and harvested 72 h later. (**C**) HepAD38 cells, in which HBV transcription is controlled by a TC-off system resulting in HBV replication, were plated with (lane 1) or without TC (lane 2) and harvested after 72 h. (**D**) HepG2 (lane 1) and HepG2.2.15 (lane 2) cells were plated, harvested after 72 h, and lysed. The lysates were subjected to SDS-PAGE and immunoblotted with primary antibodies to p-CaMKII, CaMKII, HBc, and GAPDH. To detect the core particle, lysates were subjected to 1% native agarose gel electrophoresis (NAGE) and then incubated with anti-HBc antibody. HBV DNA synthesis was analyzed by Southern blotting. HBV replicative intermediate, partially double-stranded RC, and double-stranded linear DNAs are marked as HBV RI, RC, and DL, respectively. Relative expression was quantified by normalization to GAPDH (loading control) using ImageJ 1.50b software. The level of p-CaMKII was normalized to total CaMKII expression. * *p* < 0.05, ** *p* < 0.005 relative to control by Student’s *t*-tests (*n* = 3). The bars represent means ± SD of three independent experiments.

To replicate, many viruses utilize host kinases that are involved in signaling pathways [47]. Because CaMKII activation was reported to inhibit HCC metastasis [28,29], CaMKII phosphorylation levels were analyzed in cells in which HBV was undergoing replication (Figure 1). Transient transfection of 1.3 mer WT HBV (ayw subtype) into Huh7 or HepG2 cells resulted in lower levels of activated CaMKII, as shown by T286 phosphorylation relative to control cells transfected with empty vector (Figure 1A,B, top panel, lane 1 vs. 2). The phosphorylation of CaMKII was also analyzed in HBV replicating stable HepAD38 and HepG2.2.15 cells, in which HBV was undergoing replication (Figure 1C,D). Removal of TC from HepAD38 cells, which was shown to induce HBV pgRNA transcription [36,37], also reduced the amount of active, phosphorylated CaMKII (Figure 1C, top panel, lane 1 vs. 2). Similarly, the amount of active, phosphorylated CaMKII was lower in HepG2.2.15 than in control HepG2 cells (Figure 1D, top panel, lane 1 vs. 2). These results indicate that HBV replication inhibits CaMKII activation. Because the level of total CaMKII was generally increased in HBV replicating cells (Figure 1A,C,D), phosphorylated CaMKII level normalized to total CaMKII level (p-CaMKII/total CaMKII) showed an even greater reduction in HBV replicating cells than in control cells (Figure 1 and all the following results). 

### 3.2. CaMKII and AMPK Activities Tend to Be Lower in Tumor Tissues than Adjacent Non-Tumor Tissues from Patients with HBV-Associated HCC 

**Figure 2:**
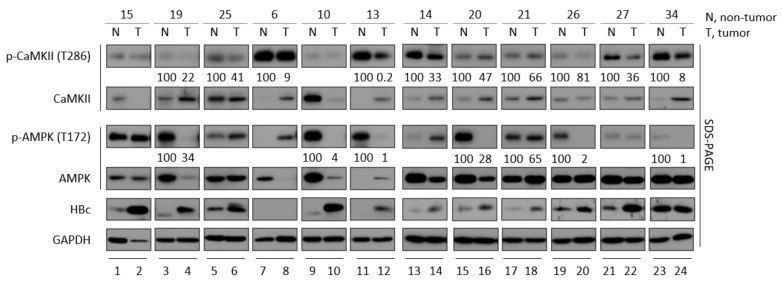
Phosphorylation and expression of CaMKII and AMPK in patients with HBV-associated hepatocellular carcinoma. Paired tumor and non-tumor liver biopsy specimens from HBV-associated HCC patients were lysed in M-PER buffer with protease and phosphatase inhibitors [3]. The lysates were subjected to SDS-PAGE and immunoblotting with primary antibodies to HBc, p-CaMKII, CaMKII, p-AMPK, and AMPK. Levels of p-CaMKII and p-AMPK were normalized to total CaMKII and AMPK levels, respectively. N, non-tumor; T, tumor.

Because the most significant risk factors for HCC development are infections with HBV and hepatitis C virus (HCV) [2], this study compared phosphorylated CaMKII levels in paired tumor and non-tumor liver biopsy specimens from twelve patients with HBV-associated HCC, who had serum HBV DNA concentrations of 6,400,000; 287,000; 939,000; 3990; 0; 48; 0; 85,800; 287; 41,200; 0; and 0 IU/mL, respectively. The clinical characteristics of HBV-associated HCC patients were presented in Table S3. Although HBV DNAs were not detected in the serum of four patients (Table S3), tissue samples from these patients were positive for HBc proteins (Figure 2, panel 5, lanes 9, 10, 13, 14 and 21–24). Although HBc proteins were expressed by both tumor and adjacent non-tumor liver tissues from eleven of these patients, the level of HBc was higher in each tumor than in the adjacent non-tumor tissue (Figure 2, panel 5, lanes 1–6 and 9–22); the exceptions were patient No. 6, who had undetectable HBc protein and patient No. 34, who had same level of HBc protein. Evaluation of eight biopsy specimens, from patients Nos. 19, 25, 13, 14, 20, 21, 26 and 27, showed that, when HBc protein level was higher, phosphorylated CaMKII was lower in tumor tissue than in paired non-tumor tissue (Figure 2, top panel, lanes 3–6 and 11–22). Interestingly, although HBc was undetectable and showed same level from patient No. 6 and No.34, respectively, the level of phosphorylated CaMKII was lower in tumor than in paired non-tumor tissue (Figure 2, top panel, lanes 7 vs. 8 and 23 vs. 24). 

Because activated AMPK restricts HBV replication via autophagic degradation [13], and CaMKII is an upstream regulator of AMPK [48,49], the level of phosphorylated AMPK was also determined (Figure 2, panel 3, lanes 3, 4, 9–12, 15–20, 23 and 24). Similar to CaMKII phosphorylation, the level of active, phosphorylated AMPK at T172 was lower in seven biopsy specimens from patients Nos. 19, 10, 13, 20, 21, 26 and 34, indicating that HBV-associated HCCs tend to inhibit the activation of CaMKII and AMPK. 

### 3.3. Effects of HBV Replication on CaMKII, AMPK, and AKT/mTOR Phosphorylation

**Figure 3:**
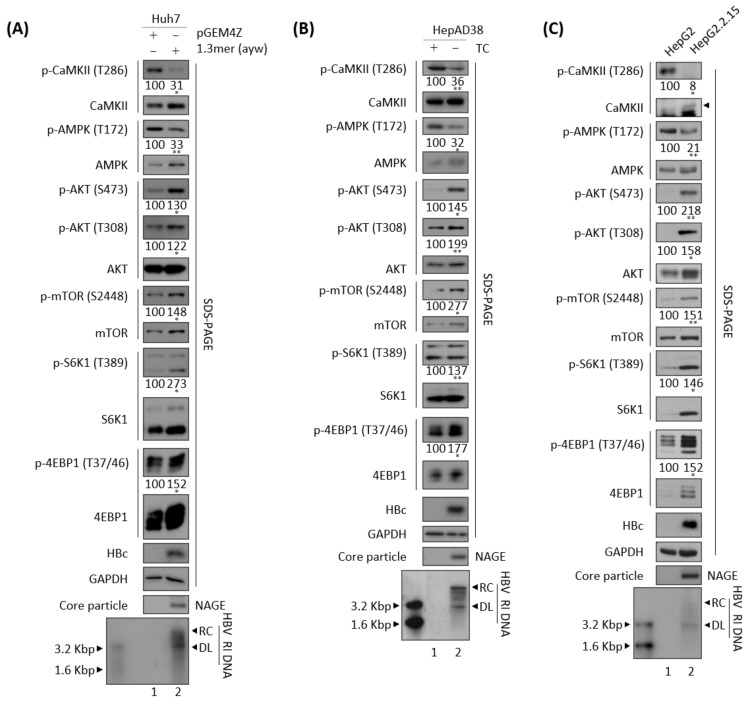
Association of HBV replication with reduced AMPK phosphorylation and increased AKT/mTOR activity. (**A**) Huh7 cells were transfected with pGEM4Z (lane 1) or 1.3 mer HBV WT (ayw) (lane 2). (**B**) HepAD38 cells were seeded with (lane 1) or without TC (lane 2). (**C**) HepG2 (lane 1) and HepG2.2.15 (lane 2) cells were seeded and incubated. Lysates were prepared 72 h after transfection (**A**), incubation in the absence of TC (**B**), and incubation (**C**), and levels of phosphorylation and expression of the indicated proteins were determined as described in Figure 1, as were core particles and HBV DNA synthesis. The relative expression of each protein was quantified by normalization to GAPDH (loading control) using ImageJ 1.50b software. * *p* < 0.05, ** *p* < 0.005 relative to control by Student’s *t*-tests (*n* = 3).

HBx has been shown to increase mTOR/S6K1 phosphorylation, thereby enhancing the development of HBV-mediated HCC [50]. Considering our findings, that active, phosphorylated CaMKII was decreased in HBV replicating cells (Figure 1), and that phosphorylated CaMKII and AMPK levels tend to be lower in tumor than in non-tumor tissue of patients with HBV-mediated HCC (Figure 2), we examined the phosphorylation of mTOR, S6K1, and 4EBP1 in HBV replicating cells (Figure 3). Consistent with the reduction in active, phosphorylated CaMKII (Figure 1 and Figure 3A–C, panels 1 and 2, and Figure S1) associated with HBV replication (bottom panels), the levels of both total and phosphorylated mTOR were increased (Figure 3A–C, panels 8 and 9), as were the levels of total and phosphorylated S6K1 and 4EBP, which act downstream of mTOR, (Figure 3A–C, panels 10–13), indicating that HBV replication enhances the mTOR/S6K1/4EBP1 signaling pathway. The levels of phosphorylated mTOR, S6K1, and 4EBP showed greater relative increases than their respective total protein levels in HBV replicating cells (Figure 3A–C). 

HBV replication activates AKT as shown by phosphorylation at S473 and T308 [3,31]. AKT inhibits AMPK [51] and activates mTOR [52]. Conversely, AMPK can also inhibit AKT [53]. Consistent with previous findings, the present study found that normalized AMPK phosphorylation at T172 (p-AMPK/total AMPK) was significantly reduced (Figure S1), and normalized AKT phosphorylation at S473 and T308 (p-AKT/total AKT) was significantly increased, in HBV replicating cells (Figure 3A–C, panels 3–7), suggesting that HBV replication affects the CaMKII/AMPK/AKT/mTOR/S6K1/4EBP1 signaling pathway.

Since total CaMKII and AMPK protein levels were increased in HBV replicating cells, we hypothesized that expressions of CaMKII and AMPK might be increased through HBx because HBx regulates the transcription of several genes. Additionally, HBV replication itself may influence several gene expressions with various mechanisms [31]. In accordance with our hypothesis, real-time RT-PCR results revealed that mRNA levels of CaMKII and AMPK were significantly increased in HBV replicating cells (Figure S2).

### 3.4. CaMKII Overexpression Inhibits the HBV Replication through AKT/mTOR Signaling

**Figure 4:**
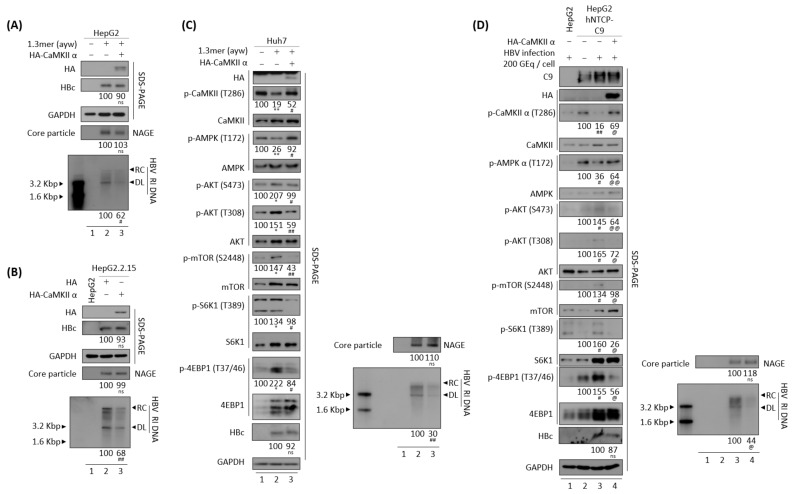
Overexpression of CaMKII inhibits HBV replication through the AKT/mTOR signaling pathway. (**A**) HepG2 cells were mock–transfected (lane 1) or co–transfected with 1.3 mer HBV WT (ayw) plus pCMV-HA (lane 2) or pCMV-HA-CaMKII (lane 3). (**B**) HepG2.2.15 cells were transfected with pCMV-HA (lane 2) or pCMV-HA-CaMKII (lane 3). Mock-transfected HepG2 cells were the negative control (lane 1). (**C**) Huh7 cells were transfected as in Figure 4A. Cells were harvested 72 h later. (**D**) HepG2 (lane 1), HepG2-hNTCP-C9 (lane 3), and CaMKII-overexpressing HepG2-hNTCP-C9 cells (lane 4) were infected with 2 × 10^2^ GEq of HBV per cell and incubated for 7 days. HepG2-hNTCP-C9 cells were mock–infected and incubated as above (lane 2). The phosphorylation and expression of the indicated proteins, core particle, and HBV DNA synthesis were analyzed as described in Figure 1. Relative expression was quantified by normalization to GAPDH (loading control) using ImageJ 1.50b software. ns, not significant; * *p* < 0.05, ** *p* < 0.005 relative to mock-transfected control by Student’s *t*-tests (lane 1 vs. 2). ^#^ *p* < 0.05, ^##^ *p* < 0.005 relative to corresponding control by Student’s *t*-tests (lane 2 vs. 3). ^@^ *p* < 0.05, ^@@^ *p* < 0.005 relative to corresponding control by Student’s *t*-tests (lane 3 vs. 4) (*n* = 3).

Because HBV replication was found to inhibit the activation of both CaMKII and AMPK (Figure 1 and Figure 3), the effects of CaMKII overexpression were evaluated in HBV replicating cells. Transient transfection of HepG2 cells with 1.3 mer HBV WT, alone or together with CaMKII α, showed that HBV DNA synthesis was decreased in the co-transfected cells (Figure 4A, bottom panel, lane 2 vs. 3). In addition, HBV DNA synthesis was lower in CaMKII-transfected HepG2.2.15 cells than in HepG2 cells (Figure 4B, bottom panel, lane 2 vs. 3). However, CaMKII overexpression did not significantly affect HBc protein expression and core particle formation (Figure 4A,B, panels 2 and 4, lane 2 vs. 3).

In accordance with above result and Figure 3, HBV transfection of Huh7 cells was found to reduce the levels of active, phosphorylated CaMKII and AMPK, and to increase the levels of active, phosphorylated AKT, mTOR, and S6K1, as well as inhibitory phosphorylated 4EBP1 (Figure 4C, panels 2–14, lane 1 vs. 2, and Figure S3A). Transfection of Huh7 cells with CaMKII α and the 1.3 mer HBV WT reduced HBV DNA synthesis (Figure 4C, bottom panel, lane 2 vs. 3). Accordingly, CaMKII and AMPK were activated; AKT, mTOR, and S6K1 were inhibited; and 4EBP1 was activated (Figure 4C, panels 2–14, lane 2 vs. 3, and Figure S3A). 

To verify the effect of CaMKII α on the HBV infection system, HepG2-hNTCP-C9 cells were established and CaMKII was overexpressed using a lentiviral vector. Briefly, CaMKII-overexpressing HepG2-hNTCP-C9 stable cells were infected with 2 × 10^2^ GEq of HBV (Figure 4D), as described previously [31,33]. As expected, CaMKII and AMPK activities were reduced, and the AKT/mTOR signaling pathway increased in these HBV-infected cells (Figure 4D, panels 3–15, lane 2 vs. 3, and Figure S3B). Upon CaMKII α overexpression, phosphorylation of AMPK was increased and phosphorylation of AKT/mTOR was decreased, resulting in the downregulation of HBV DNA synthesis (Figure 4D, lane 3 vs. 4, and Figure S3B). Taken together, these results demonstrate that CaMKII α overexpression represses HBV replication by activating AMPK and inhibiting the AKT/mTOR signaling pathway, indicating that CaMKII α is an upstream protein in the AMPK/AKT/mTOR signaling pathway in HBV replicating cells.

### 3.5. AMPK Overexpression or Activation Inhibits HBV Replication

**Figure 5:**
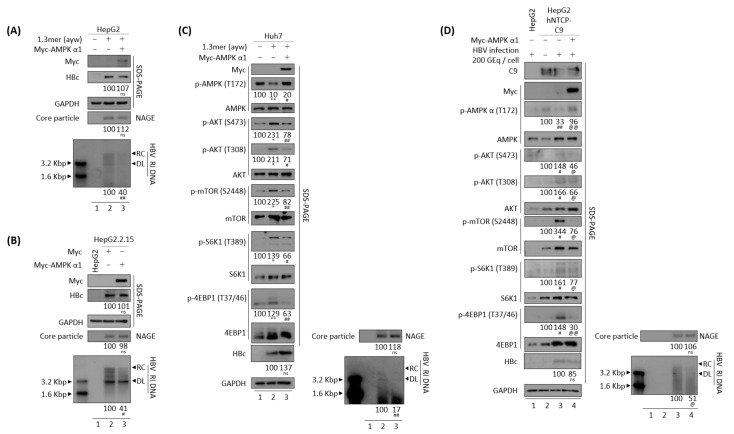
Overexpression of AMPK reduces HBV replication through the AKT/mTOR signaling pathway. (**A**) HepG2 cells were mock transfected (lane 1) or co–transfected with 1.3 mer HBV WT (ayw) plus pCMV-Myc (lane 2) or pCMV-Myc-AMPK (lane 3). (**B**) HepG2.2.15 cells were transfected with pCMV-Myc (lane 2) or pCMV-Myc-AMPK (lane 3). Mock-transfected HepG2 cells were the negative control (lane 1). (**C**) Huh7 cells were transfected as above in (A). Cells were harvested 72 h after transfection. (**D**) HepG2 (lane 1), HepG2-hNTCP-C9 (lane 3), and AMPK overexpressing HepG2-hNTCP-C9 cells (lane 4) were infected as described in Figure 4D. The phosphorylation and expression of the indicated proteins, core particles, and HBV DNA synthesis were analyzed as described in Figure 1. Relative expression was quantified by normalization to GAPDH (loading control) using ImageJ 1.50b software. ns, not significant; * *p* < 0.05, ** *p* < 0.005 relative to mock-transfected control by Student’s *t*-tests (lane 1 vs. 2). ^#^ *p* < 0.05, ^##^ *p* < 0.005 relative to corresponding control by Student’s *t*-tests (lane 2 vs. 3). ^@^ *p* < 0.05, ^@@^ *p* < 0.005 relative to corresponding control by Student’s *t*-tests (lane 3 vs. 4) (*n* = 3).

Since AMPK seems to act downstream of CaMKII (Figure 4C,D) [48,49], the effect of AMPK overexpression was analyzed in HBV replicating cells. Similar to overexpression of CaMKII α in HBV replicating cells (Figure 4), AMPK α1 overexpression reduced HBV DNA synthesis in transiently co-transfected HepG2 and Huh7 cells (Figure 5A,C, bottom panel, lane 2 vs. 3), and in AMPK α1-transfected stable HepG2.2.15 cells (Figure 5B, bottom panel, lane 2 vs. 3). Furthermore, overexpression of AMPK induced the dephosphorylation of AKT, mTOR, S6K1, and 4EBP1 (Figure 5C, panels 4–12, lane 2 vs. 3, and Figure S3C). By contrast, HBc protein expression and core particle formation were not affected by AMPK overexpression (Figure 5A–C, lane 2 vs. 3).

To explore the effect of AMPK α1 on HBV-infected cells, AMPK was overexpressed in HepG2-hNTCP-C9 cells using a lentiviral vector. Briefly, AMPK-overexpressing HepG2-hNTCP-C9 stable cells were infected with 2 × 10^2^ GEq of HBV (Figure 5D), as described above. As expected, AMPK phosphorylation was inhibited and AKT/mTOR phosphorylation increased in HBV-infected cells (Figure 5D, panels 3–13, lane 2 vs. 3). Similar to findings in AMPK-overexpressing, HBV-transfected cells (Figure 5C), the levels of phosphorylated AKT, mTOR, S6K1, and 4EBP1 were downregulated in AMPK-overexpressing, HBV-infected HepG2-hNTCP-C9 cells, resulting in decreased HBV DNA synthesis (Figure 5D, lane 3 vs. 4, and Figure S3D). These results demonstrate that AMPK α1 overexpression inhibits HBV replication by inhibiting the AKT/mTOR signaling pathway.

Because the AMPK activator metformin inhibits HBV replication [12] and AMPK overexpression inhibits HBV replication (Figure 5), the effect of AMPK activation was further examined in HBV replicating cells. Treatment of HepG2.2.15 and HepAD38 cells with 2 or 4 mM metformin for 24 h increased AMPK phosphorylation and decreased the levels of HBc protein and core particles, as well as HBV DNA synthesis (Figure S5A,C, lane 2 vs. 3 and 4, lane 3 vs. 4 and 5). Treatment of these cells with 1 or 2 mM AICAR, another AMPK activator, for 24 h also decreased the levels of HBc protein and core particles, as well as HBV DNA synthesis (Figure S5B,C, lane 2 vs. 3 and 4, lane 6 vs. 7 and 8). MTT assay results revealed that metformin and AICAR were not cytotoxic to HepG2.2.15 and HepAD38 cells (Figure S4A,B). Treatment of cells with 4 mM metformin or 2 mM AICAR significantly reduced the levels of HBc protein and core particles, as well as HBV DNA synthesis (Figure S5, lane 2 vs. 4, lane 6 vs. 8), with both agents having a greater effect on HBV DNA (Figure S5, bottom panels) than on the levels of HBc protein and core particles (Figure S5, panels 3 and 5). Taken together, these findings indicate that AMPK overexpression (Figure 5) and activation (Figure S5) have negative effects on HBV replication.

### 3.6. Inhibition of CaMKII Enhances HBV Replication 

**Figure 6:**
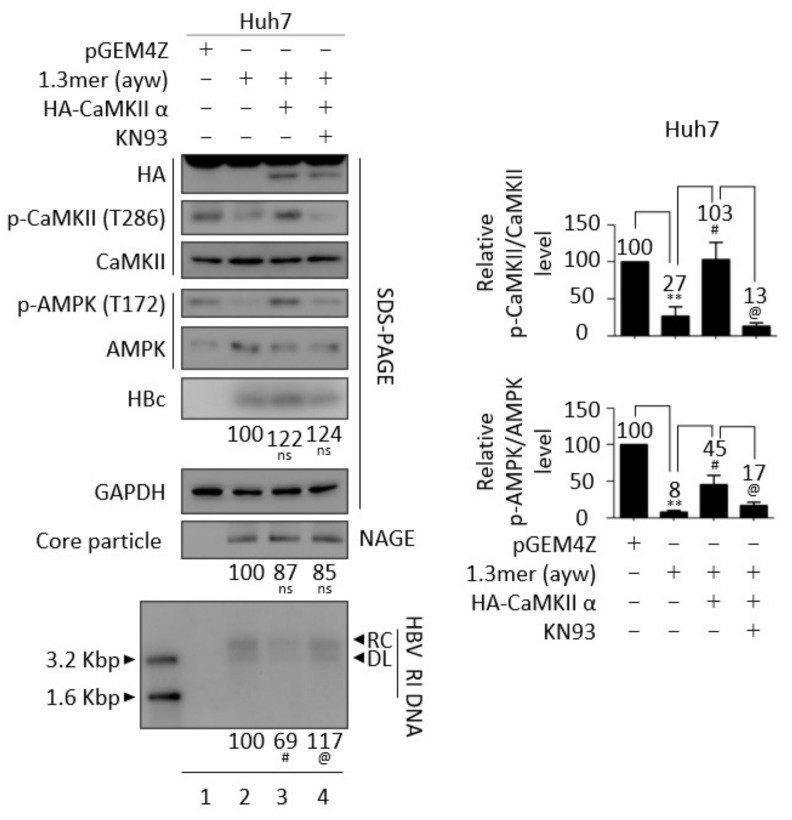
Inhibition of CaMKII enhances HBV replication. Huh7 cells were transfected with pGEM4Z (lane 1) or 1.3 mer HBV WT (ayw) (lane 2) or co–transfected with 1.3 mer HBV WT plus pCMV-HA-CaMKII (lanes 3 and 4). After 24 h, co-transfected cells were treated with DMSO (lane 3) or 10 μM KN93 (lane 4), a CaMKII inhibitor, for 48 h and harvested. Phosphorylation and expression of indicated proteins, core particles, and HBV DNA synthesis were analyzed as described in Figure 1. Relative expression was quantified by normalization to GAPDH (loading control) using ImageJ 1.50b software. ns, not significant; ** *p* < 0.005 relative to control by Student’s *t*-tests (lane 1 vs. 2). ^#^ *p* < 0.05 relative to corresponding control by Student’s *t*-tests (lane 2 vs. 3). ^@^ *p* < 0.05 relative to corresponding control by Student’s *t*-tests (lane 3 vs. 4) (*n* = 3). The bars represent means ± SD of three independent experiments.

Because overexpression of CaMKII upregulated AMPK activity and downregulated the AKT/mTOR signaling pathway in HBV replicating cells (Figure 4C,D), the effects of the CaMKII inhibitor KN93 on the AMPK/AKT/mTOR signaling pathway were evaluated. However, because CaMKII activity is downregulated in HBV replicating cells (Figure 1, Figure 3 and Figure 4C,D), CaMKII was overexpressed before treatment with the CaMKII inhibitor. Although Huh7 cells transiently co-transfected with 1.3 mer HBV WT plus CaMKII showed increased AMPK activity, inhibition of the AKT/mTOR signaling pathway, and downregulation of HBV DNA synthesis (Figure 6 and Figure S6, lane 2 vs. 3), treatment of these cells with 10 μM KN93 inhibited CaMKII and subsequent AMPK phosphorylation, while restoring the AKT/mTOR signaling pathway and increasing HBV DNA synthesis (Figure 6 and Figure S6, lane 3 vs. 4). There was no significant cell viability change in 10μM KN93-treated Huh7 cells (Figure S4C, top panel). The findings, that HBV replication inhibits CaMKII and AMPK activations and activates AKT/mTOR signaling, and that CaMKII overexpression inhibits HBV replication while inhibiting AKT/mTOR signaling, suggest that CaMKII may regulate the AMPK/AKT/mTOR signaling pathway in HBV replicating cells. 

### 3.7. CaMKII and AMPK Form a Feedback Loop

**Figure 7:**
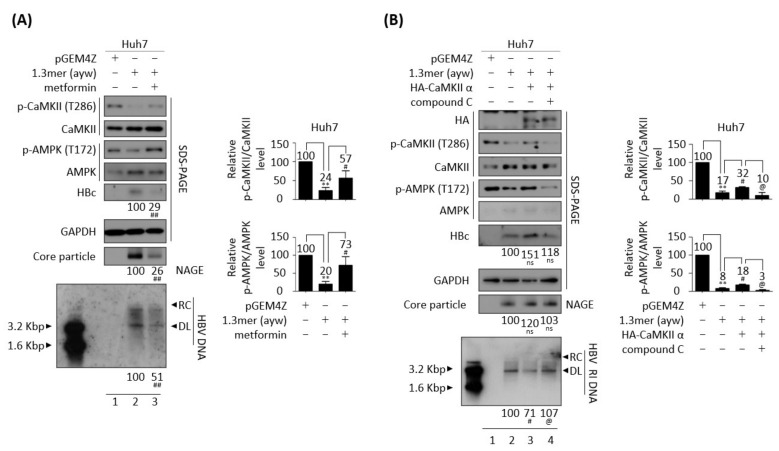
Effect of AMPK activator and inhibitor on CaMKII phosphorylation during HBV replication. (**A**) Metformin, an AMPK activator, also activates CaMKII in Huh7 cells. Huh7 cells were transiently transfected with pGEM4Z (lane 1) or 1.3 mer HBV WT (ayw) (lanes 2 and 3) and incubated with distilled water (lane 2) or 2 mM metformin (lane 3) for 48 h. (**B**) Compound C, an AMPK inhibitor, also inhibits CaMKII in CaMKII-overexpressing Huh7 cells. Huh7 cells were transiently transfected with pGEM4Z (lane 1) or 1.3 mer HBV WT (ayw) (lane 2) or co–transfected with 1.3 mer HBV WT (ayw) plus pCMV–HA–CaMKII (lanes 3 and 4). Co-transfected cells were treated with DMSO (lane 3) or 5 μM compound C (lane 4) for 48 h. Phosphorylation and expression of the indicated proteins, core particles, and HBV DNA synthesis were analyzed as described in Figure 1. Relative expression was quantified by normalization to GAPDH (loading control) using ImageJ 1.50b software. Student’s *t*-test. ns, not significant; ** *p* < 0.005 relative to control by Student’s *t*-tests (lane 1 vs. 2). ^#^ *p* < 0.05, ^##^ *p* < 0.005 relative to corresponding control by Student’s *t*-tests (lane 2 vs. 3). ^@^ *p* < 0.05 relative to corresponding control by Student’s *t*-tests (lane 3 vs. 4) (*n* = 3). The bars represent means ± SD of three independent experiments.

**Figure 8:**
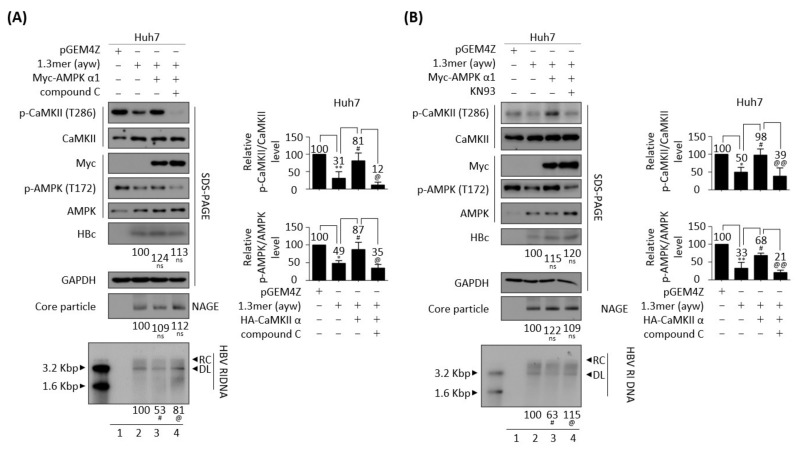
CaMKII-AMPK form a positive feedback loop. (**A**) Compound C inhibits both AMPK and CaMKII in AMPK-overexpressing Huh7 cell. Huh7 cells were transiently transfected with pGEM4Z (lane 1) or 1.3 mer HBV WT (ayw) (lane 2) or co-transfected with 1.3 mer HBV WT (ayw) plus pCMV-Myc-AMPK (lanes 3 and 4). Co-transfected cells were treated with DMSO (lane 3) or 5 μM compound C (lane 4) for 48 h. (**B**) KN93, a CaMKII inhibitor, inhibits both AMPK and CaMKII in AMPK-overexpressing Huh7 cells. Huh7 cells were (co-)transfected as in (A) and treated with DMSO (lane 3) or 10 μM KN93 for 48 h. The phosphorylation and expression of the indicated proteins, core particles, and HBV DNA synthesis were analyzed as described in Figure 1. Relative expression was quantified by normalization to GAPDH (loading control) using ImageJ 1.50b software. ns, not significant; * *p* < 0.05, ** *p* < 0.005 relative to control by Student’s *t*-tests (lane 1 vs. 2). ^#^ *p* < 0.05 relative to corresponding control by Student’s *t*-tests (lane 2 vs. 3). ^@^ *p* < 0.05, ^@@^ *p* < 0.005 relative to corresponding control by Student’s *t*-tests (lane 3 vs. 4) (*n* = 3). The bars represent means ± SD of three independent experiments.

Because metformin and AICAR repressed HBV replication (Figure S5), the effect of metformin on the CaMKII/AMPK/AKT signaling pathway was evaluated in HBV replicating cells. Consistent with the above results, treatment of 1.3 mer HBV WT-transfected Huh7 cells with 2 mM metformin inhibited the AKT/mTOR signaling pathway by enhancing AMPK activation, while significantly reducing HBc protein expression, core particle formation, and HBV DNA synthesis (Figure 7A and Figure S7A, lane 2 vs. 3). 2mM metformin was not cytotoxic to Huh7 cell (Figure S4C, middle panel). Although CaMKII acts upstream of AMPK (Figure 4C,D) [48,49], CaMKII phosphorylation was also increased by metformin (Figure 7A, top panel, lane 2 vs. 3). Because AMPK activation induces CaMKII activation (Figure 7A), we hypothesized that CaMKII and AMKP may form a feedback loop, thereby affecting the AKT/mTOR signaling pathway during HBV replication. To further evaluate this hypothesis, Huh7 cells co-transfected with 1.3 mer HBV WT plus CaMKII (Figure 7B and Figure S7B, lanes 3 and 4) were treated with the AMPK inhibitor compound C (5 μM) (Figure 7B and Figure S7B, lane 4), which reduced the activities of CaMKII and AMPK and enhanced AKT/mTOR activities, resulting in increased HBV DNA synthesis (Figure 7B, lane 3 vs. 4). HBc expression and core particle formation, however, were not changed significantly. Cell viability was not affected by 5μM compound C treatment to Huh7 cells (Figure S4C, bottom panel). To further verify the hypothesis in AMPK-overexpressing cells, Huh7 cells co-transfected with 1.3 mer HBV WT plus AMPK were treated with compound C and the CaMKII/AMPK/AKT signaling pathway was evaluated (Figure 8A and Figure S7C). AMPK overexpression was found to increase CaMKII activity in these cells, reducing HBV DNA synthesis by suppressing the AKT/mTOR signaling pathway (Figure 8A and Figure S7C, lane 2 vs. 3). The AMPK inhibitor also blocked CaMKII phosphorylation and activated the AKT/mTOR signaling pathway, increasing HBV DNA synthesis (Figure 8A and Figure S7C, lane 3 vs. 4). 

Huh7 cells co-transfected with 1.3 mer HBV WT plus AMPK were treated with the CaMKII inhibitor KN93 (Figure 8B and Figure S7D, lane 4) to examine the effect of KN93 on the CaMKII/AMPK/AKT/mTOR signaling pathway. AMPK-co-transfection was found to activate CaMKII/AMPK and suppress the AKT/mTOR signaling pathway, thereby reducing HBV DNA synthesis (Figure 8B and Figure S7D, lane 2 vs. 3). Similar to compound C, KN93 blocked CaMKII/AMPK activation, inhibited the AKT/mTOR signaling pathway, and increased HBV replication (Figure 8B and Figure S7D, lane 3 vs. 4). These results indicate that CaMKII and AMKP may form a positive feedback loop for HBV replication. 

### 3.8. CaMKII Overexpression Reduces HBV RNAs Due to the Decreased HBV cccDNA 

**Figure 9:**
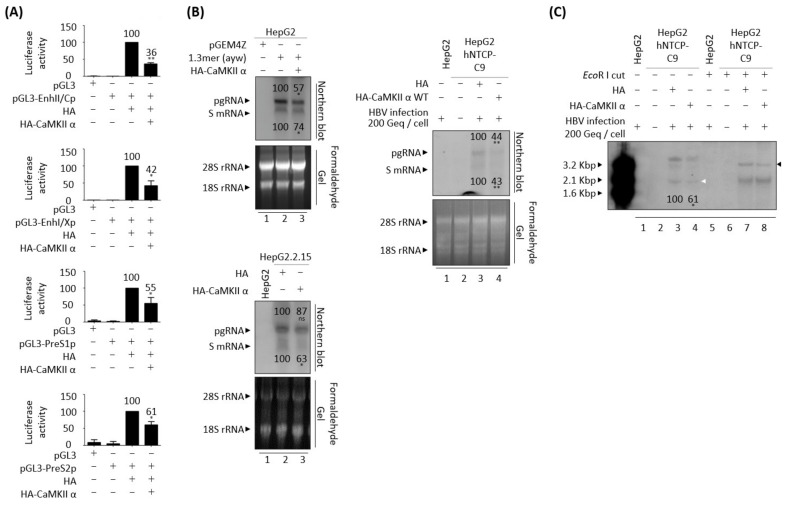
CaMKII overexpression reduces HBV cccDNA, decreasing HBV RNA transcription and RNA level. (**A**) Luciferase reporter assays of HBV enhancer and promoter activities in response to CaMKII overexpression. HepG2 cells were transiently transfected with the indicated luciferase reporter vectors in the presence or absence of pCMV-HA-CaMKII. (**B**) Northern blotting, showing the decreased expression of HBV mRNAs upon overexpression of CaMKII. (Upper left) HepG2 cells were transiently mock transfected (lane 1) or co-transfected with 1.3 mer HBV WT (ayw) plus pCMV-HA (lane 2) or pCMV-HA-CaMKII (lane 3). (Lower left) HepG2.2.15 cells were transfected with pCMV-HA (lane 2) or pCMV-HA-CaMKII (lane 3). HepG2 cell was a negative control (lane 1). (Right) HepG2 (lane 1), HepG2-hNTCP-C9 (lanes 2 and 3), and CaMKII-overexpressing HepG2-hNTCP-C9 cells (lane 4) were infected as described in Figure 4D. Total RNA was harvested at 72 h after transfection. Northern blotting was performed as described [41]. (**C**) HBV cccDNA levels are decreased upon overexpression of CaMKII in HBV-infected cells. Cells were infected as described in Figure 4D. The white and black arrowheads indicate cccDNA and linearized cccDNA to a genome-length DL DNA, respectively. ns, not significant; * *p* < 0.05, ** *p* < 0.005 relative to control by Student’s *t*-tests (*n* = 3). The bars represent means ± SD of three independent experiments.

Since HBV DNA synthesis was decreased in CaMKII-overexpressing cells (Figure 4, Figure 6 and Figure 7B), HBV transcription was evaluated in CaMKII-overexpressing HepG2 cells using luciferase reporter assay (Figure 9A). Overexpression of CaMKII reduced transcriptional activities of enhancer II/core promoter (EnhII/Cp), preS1 promoter, preS2 promoter, and enhancer I/X promoter (EnhI/Xp) (Figure 9A). Northern blotting showed that the levels of HBV pgRNA and subgenomic S mRNAs were significantly reduced in HepG2 cells co-transfected with 1.3 mer HBV WT plus CaMKII and in HBV-infected CAMKII-overexpressing HepG2-hNTCP-C9 cells (Figure 9B, upper left and right). Subgenomic S mRNA was significantly reduced in CaMKII-transfected-HepG2.2.15 cells, but pgRNA level was not (Figure 9B, lower left). These findings suggest that the downregulation of HBV transcription in CaMKII-overexpressing cells reduced the levels of HBV RNAs. In addition, HBV cccDNA was significantly reduced in HBV-infected cells (Figure 9C, lane 3 vs. 4, white arrowhead, and Figure S8A). Taken together, these results indicated that CaMKII overexpression downregulates HBV cccDNA, resulting in decreased syntheses of HBV RNAs and RI DNAs.

### 3.9. AMPK Overexpression Reduces HBV RNAs Due to the Decreased HBV cccDNA 

**Figure 10:**
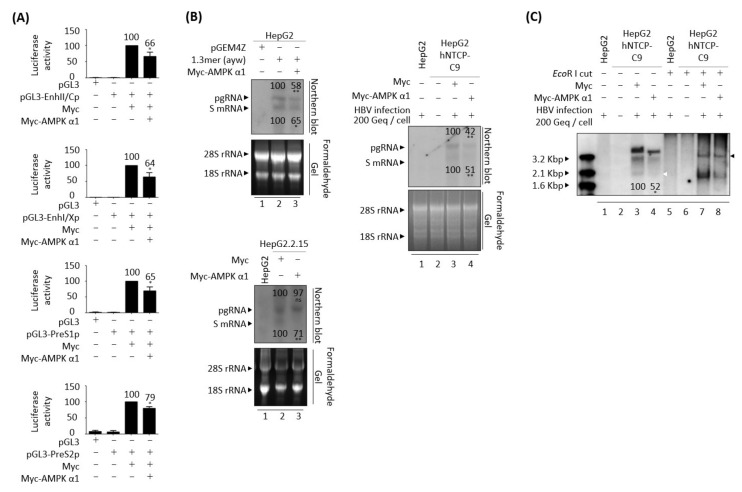
AMPK overexpression reduces HBV cccDNA, reducing HBV RNA transcription and RNA level. (**A**) Luciferase reporter assays, performed as described in Figure 9A. (**B**) Northern blotting showing reduced expression of HBV mRNAs upon overexpression of AMPK, as described in Figure 9B. (**C**) Decreased HBV cccDNA levels upon overexpression of AMPK in HBV-infected cells. ns, not significant; * *p* < 0.05, ** *p* < 0.005 relative to control by Student’s *t*-tests (*n* = 3). The bars represent means ± SD of three independent experiments.

Since either overexpression or activation of AMPK had a negative effect on HBV DNA synthesis (Figure 5, Figure S5, Figure 7A and Figure 8), HBV transcriptional activities in AMPK-overexpressing HepG2 cells were evaluated using luciferase reporter assay (Figure 10A). Similar to overexpression of CaMKII (Figure 9A), overexpression of AMPK reduced the transcriptional activities of enhancer II/core promoter (EnhII/Cp), preS1 promoter, preS2 promoter, and enhancer I/X promoter (EnhI/Xp) (Figure 10A). 

Northern blotting showed the levels of HBV pgRNA and subgenomic S mRNAs were reduced in HepG2 cells co-transfected with 1.3 mer HBV WT plus AMPK and in HBV-infected AMPK-overexpressing HepG2-hNTCP-C9 cells (Figure 10B, upper left and right). Subgenomic S mRNA was significantly reduced in AMPK transfected-HepG2.2.15 cells, whereas pgRNA level was not (Figure 10B, lower left). HBV cccDNA level was also significantly reduced in HBV-infected, AMPK-overexpressing HepG2-hNTCP-C9 cells, suggesting that HBV cccDNA was decreased by AMPK overexpression in HBV-infected cells (Figure 10C, lane 3 vs. 4, white arrowhead, and Figure S8B). Similar to findings in CaMKII-overexpressing cells, these results indicate that AMPK overexpression downregulates HBV cccDNA, reducing the synthesis of HBV RNAs and RI DNAs.

### 3.10. Reduced HBV Replication by Overexpression of CaMKII or AMPK Is HBx Independent

**Figure 11:**
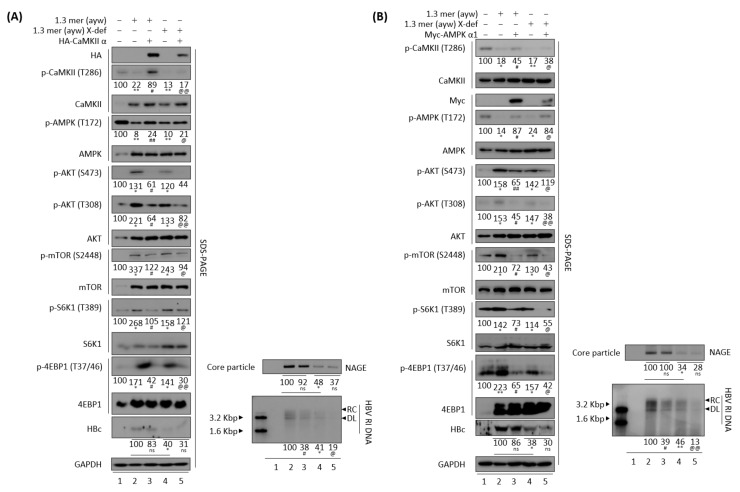
Reduced HBV replication induced by CaMKII or AMPK overexpression is independent of HBx. (**A**) In the presence or absence of HBx, CaMKII overexpression decreases HBV replication by activating CaMKII and AMPK, and by inhibiting the AKT/mTOR signaling pathway. (**B**) In the presence or absence of HBx, AMPK overexpression decreases HBV replication by activating CaMKII and AMPK, and by inhibiting the AKT/mTOR signaling pathway. ns, not significant; * *p* < 0.05, ** *p* < 0.005 relative to control by Student’s *t*-tests (lane 1 vs. 2 or 4). ^#^ *p* < 0.05, ^##^ *p* < 0.005 relative to corresponding control by Student’s *t*-tests (lane 2 vs. 3). ^@^ *p* < 0.05, ^@@^ *p* < 0.005 relative to corresponding control by Student’s *t*-tests (lane 4 vs. 5) (*n* = 3).

Because overexpression of CaMKII significantly reduced the transcriptional activity of enhancer I and HBx promoter (Figure 9A, second panel), and because many HBV-associated signaling pathways depend on HBx [50], we assessed whether this reduction in HBV replication was affected by HBx. HBV replication was lower in HepG2 cells transiently transfected with HBx-deficient mutant HBV than with HBV WT [54] (Figure 11A, panels 15, 17, and bottom, lane 2 vs. 4), as was activated AKT/mTOR signaling (Figure 11A, panels 6–14, lane 2 vs. 4), although CaMKII/AMPK activities were similar (Figure 11A, panels 2–5, lane 2 vs. 4). Interestingly, HBV replication was further decreased upon co-transfection of HBx-deficient mutant plus CaMKII α, whereas AMPK phosphorylation was increased and AKT/mTOR signaling was decreased (Figure 11A, lane 4 vs. 5). These findings indicated that the AMPK/AKT/mTOR signaling pathway and the reduction of HBV DNA synthesis induced by CaMKII overexpression were independent of HBx (Figure 11A, lane 4 vs. 5).

HBx has been shown to promote lipid accumulation and hepatic steatosis through a transcriptional activation and signaling pathway associated with metabolism [55,56]. Since HBx regulates intracellular ATP and the AMPK signaling pathway to facilitate persistent HBV replication [57], and sustains HCC cell survival [58], we evaluated whether repression of HBV replication through AMPK overexpression was dependent on HBx. HepG2 cells were therefore transiently co-transfected with AMPK α1 plus 1.3 mer HBV WT or HBx-deficient HBV mutant. As expected, HBV replication was suppressed in cells co-transfected with HBV WT plus AMPK α1 (Figure 11B, panels 15, 17, and bottom, lane 2 vs. 3) through an increased CaMKII phosphorylation (Figure 11B, top panel, lane 2 vs. 3) due to a CaMKII–AMPK feedback loop (Figure 7 and Figure 8), triggering the inhibition of AKT/mTOR activities (Figure 11B, panels 6–14, lane 2 vs. 3). The reduced HBV replication in cells transfected with HBx-deficient mutant [54] (Figure 11B, panels 15, 17, and bottom, lane 2 vs. 4) resulted in weaker activation of AKT/mTOR signaling than in cells transfected with HBV WT (Figure 11B, panels 6–14, lane 2 vs. 4), although CaMKII/AMPK activities were comparable in these cells (Figure 11B, panels 1–5, lane 2 vs. 4). HBV replication was lower, CaMKII phosphorylation was higher, and AKT/mTOR signaling was decreased in cells co-transfected with HBx-deficient mutant plus AMPK α1 than in cells transfected with HBx-deficient mutant (Figure 11B, lane 4 vs. 5). These results demonstrated that AMPK-mediated downregulation of HBV replication does not depend on HBx. 

## 4. Discussion

Chronic HBV infection is one of the main risk factors for progression of HCC [9,10,11] and HCC is the most frequent type of primary liver cancer causing cancer-related deaths worldwide [9,10,11]. Several kinases have been found to play important roles in HCC progression [28,29] and to be associated with HBV replication [12,13,14,15]. Activated CaMKII suppresses the migration of liver cancer cells by increasing intracellular calcium, thus inhibiting metastasis [28,29]. Our finding, that CaMKII phosphorylation was decreased in HBV replicating cells (Figure 1, Figure 3, Figure 4C,D, Figure 6, Figure 7, Figure 8 and Figure 11), indicates that HBV replication could enhance HCC progression by suppressing CaMKII activity. Furthermore, the activities of CaMKII and AMPK tend to be lower in HBV-associated HCCs than in paired non-tumor tissues (Figure 2). 

Because CaMKII acts upstream of AMPK [48,49], AMPK phosphorylation was also reduced in HBV replicating cells. Consistent with results showing that activated AMPK inhibits HBV replication [13], the present study found that both activated (Figure S5 and Figure 7A) and overexpressed AMPK (Figure 5, Figure 8 and Figure 11B) suppressed HBV replication. The finding, that AMPK downregulation is associated with HCC proliferation [59], suggested that the AMPK activator metformin could inhibit HCC growth [60]. Similar to downregulation of CaMKII in HBV replicating cell (Figure 1, Figure 3, Figure 4C,D, Figure 6, Figure 7, Figure 8 and Figure 11), we suggest that suppressed AMPK in HBV replicating cell (Figure 3, Figure 4C,D, Figure 5C,D, Figure 6, Figure 7, Figure 8 and Figure 11) could enhance HCC progression, whereas overexpression of either CaMKII (Figure 4C,D, Figure 6, Figure S6, Figure 7B and Figure S7B) and AMPK (Figure 5C,D, Figure 8 and Figure S7C,D) could inhibit the AKT/mTOR signaling pathway in an HBx-independent manner (Figure 11). The finding, that HBV replication activates AKT [3,14,15,31], indicated that HCC tumorigenesis and metastasis [61] could be induced by activating the AKT/mTOR signaling pathway. 

We previously showed that HBV replication upregulated the expression of Sirtuin 2 isoform 1 (Sirt2.1), strengthened AKT–Sirt2.1 interactions, and activated the AKT signaling pathway [3]. By contrast, the interaction between AKT and Sirt2 isoform 5 (Sirt2.5) was weakened by HBV replication [3]. Interactions of Sirt2 with liver kinase B (LKB1), another major upstream kinase of AMPK, results in deacetylation of LKB1, with deacetylated LKB1 enhancing AMPK activation [62]. Further studies are needed, however, to evaluate the interactions of AMPK with Sirt2.1 and Sirt2.5. AMPK was shown to phosphorylate Sirt2 at T101 through AKT–Sirt2 interactions, regulating the insulin signaling pathway [63]. The findings, that AMPK inhibits AKT to suppress cell survival and induce apoptosis [53], and that AMPK regulates AKT–Sirt2 interactions [63], suggest the need to investigate the relationships between AMPK, Sirt2, and AKT. 

Although HBV DNA synthesis was decreased in cells transfected with CaMKII (Figure 4, Figure 6, Figure 7B and Figure 11A) and AMPK (Figure 5, Figure 8 and Figure 11B), HBc protein expression and core particle formation were not significantly decreased. The C-terminal domain (CTD) of HBc protein undergoes a dynamic phosphorylation–dephosphorylation cycle during HBV replication with overlapping consensus R-x-x-S/T, R-R-x-S/T, and S/T-P phosphorylation motifs [64,65]. The phosphorylated CTD of HBc contributes to its functions, including core particle stability, pgRNA encapsidation, minus-strand and plus-strand DNA synthesis, and RC DNA synthesis [66,67]. Hyperphosphorylation of the HBc CTD by kinases neutralizes its positive charge, reducing RNA binding capacity and resulting in empty capsids [68,69]. Therefore, we hypothesized that overexpression of CaMKII or AMPK might regulate the phosphorylation of HBc protein to induce the production of empty capsids, thus decreasing HBV DNA synthesis. The phosphorylation consensus motifs for CaMKII and AMPK are R-x-x-S/T [70,71] and B-Hyd-B-x-x-x-S/T-x-x-x-Hyd [72], respectively, where Hyd represent a hydrophobic, x any, and B a basic amino acid residue. CaMKII can participate in HBc CTD phosphorylation because HBc CTD has three R-x-x-S/T motifs. AMPK may participate in HBc CTD phosphorylation indirectly. Additional studies are required to confirm this hypothesis. 

Our finding, that cccDNA was reduced in CaMKII- and AMPK-overexpressing HBV replicating cells (Figure 9C, Figure 10C and Figure S8), suggested that RNA transcription should also be decreased (Figure 9A and Figure 10A), resulting in reduced pgRNA and subgenomic S mRNA levels (Figure 9B and Figure 10B). However, the present study found that pgRNA levels were not significantly reduced in HepG2.2.15 cells transfected with CaMKII or AMPK (Figure 9B and Figure 10B, lower left). Given that HepG2.2.15 cells are HBV replicating stable cells [34,35], suggesting that HBV pgRNA levels may not be significantly reduced by CaMKII or AMPK overexpression. 

Because a complete cure of HBV infection requires the elimination of cccDNA from the cell nucleus [3], understanding the molecular mechanisms responsible for cccDNA stability is necessary to completely eradicate HBV. Since CaMKII overexpression decreases cccDNA level (Figure 9C and Figure S8A) and CaMKII α contains a nuclear localization signal (NLS) [73,74], further studies are needed to determine the intracellular localization of CaMKII α and its indirect association with cccDNA. We hypothesized that the decreased levels of HBV RNAs in cells overexpressing CaMKII (Figure 9A,B) were due to decreased levels of cccDNA (Figure 9C and Figure S8A), the template for HBV RNA transcription [75]. Furthermore, CaMKII overexpression might affect the recruitment of host RNA polymerase II or transcriptional activators or repressors, suggesting the need to evaluate the transcriptional activities of cccDNA in CaMKII-overexpressing HBV replicating cells. Because AMPK overexpression decreases cccDNA (Figure 10C and Figure S8B); HBV infection induces the accumulation of ROS [76]; and increased ROS enhances the localization of AMPK to the nucleus [77], additional studies are needed to explore the cellular localization of AMPK and the recruitment of host RNA polymerase II or transcriptional activators or repressors in AMPK-overexpressing HBV replicating cells.

Since aged liver showed the increased persistent inflammation and activated NF-κB signaling, aging is one of the most significant risk factors for chronic liver diseases [78]. Accordingly, when aged people are infected with HBV or HCV, they are more prone to be chronically infected than young adults [79]. Since aged people have declined metabolism and functional capacities, such as decreased AMPK activity and impaired insulin sensitivity, AMPK activation may promote metabolic health and prevent age-related diseases [80]. Therefore, we hypothesize that AMPK overexpression or activation may have therapeutic effects in treating not only CHB but also metabolic diseases, including obesity and type 2 diabetes, suggesting multiple effects by modulation of AMPK signaling pathway. Further studies are needed to evaluate the relationship with HBV replication, age, and AMPK/AKT/mTOR signaling pathway.

## 5. Conclusions

The present study showed that HBV replication inhibits CaMKII activation and that CaMKII overexpression reduces HBV replication via the AMPK/AKT/mTOR signaling pathway. The findings, that overexpressed CaMKII or AMPK reduced cccDNA level, inhibiting the synthesis of HBV RNAs and RI DNA, suggested that activation or overexpression of CaMKII or AMPK might be a possible therapeutic option to cure HBV infection and HBV-associated hepatocarcinogenesis. 

## Data Availability

Data are available from the corresponding author upon reasonable request.

## Supplementary Materials

The following supporting information can be downloaded at: https://www.mdpi.com/article/10.3390/microorganisms10030498/s1, Figure S1: Relative levels of total and active CaMKII and AMPK in HBV replicating cells presented in Figure 3; Figure S2: Quantitative real-time RT-PCR results to show the levels of CaMKII and AMPK mRNAs in HBV replicating cells; Figure S3: Relative levels of total and active CaMKII and AMPK in HBV replicating cells presented in Figure 4C–D and Figure 5C–D; Figure S4: Cytotoxic effects of metformin, AICAR, KN93, and compound C in indicated cells; Figure S5: Activation of AMPK reduces HBV replication; Figure S6: AKT-mTOR/S6K1/4EBP1 signaling pathway presented in Figure 6; Figure S7: AKT-mTOR/S6K1/4EBP1 signaling pathway presented in Figure 7A,B and Figure 8C,D; Figure S8: Quantitative real-time PCR of HBV cccDNA in HBV-infected cells from Figure 9C and Figure 10C; Figure S9: Uncut scans of original western blotting images of Figure 1; Figure S10: Uncut scans of original western blotting images of Figure 2; Figure S11: Uncut scans of original western blotting images of Figure 3; Figure S12: Uncut scans of original western blotting images of Figure 4; Figure S13: Uncut scans of original western blotting images of Figure 5; Figure S14: Uncut scans of original western blotting images of Figure S5; Figure S15: Uncut scans of original western blotting images of Figure 6 and Figure S6; Figure S16: Uncut scans of original western blotting images of Figure 7, Figures S7A and S7B; Figure S17: Uncut scans of original western blotting images of Figure 8, Figures S7C and S7D; Figure S18: Uncut scans of original western blotting images of Figure 11; Table S1: Primers for construction of CaMKII α and AMPK α1 expression plasmids, qPCR, and RT-qPCR; Table S2: Antibodies used for this study; Table S3: Clinical characteristics of HBV-associated HCC patients presented in Figure 2.
