# Supplementary material for: Ca2+/Calmodulin-Dependent Protein Kinase II Inhibits Hepatitis B Virus Replication from cccDNA via AMPK Activation and AKT/mTOR Suppression
Source: Microorganisms. 2022 Feb 23;10(3):498. doi: 10.3390/microorganisms10030498 (PMC8950817; doi:10.3390/microorganisms10030498)
Supplement: Supplementary file 1 [file microorganisms-10-00498-s001.zip › microorganisms-1552084-supplementary.pdf]

microorganisms

Supplementary materials

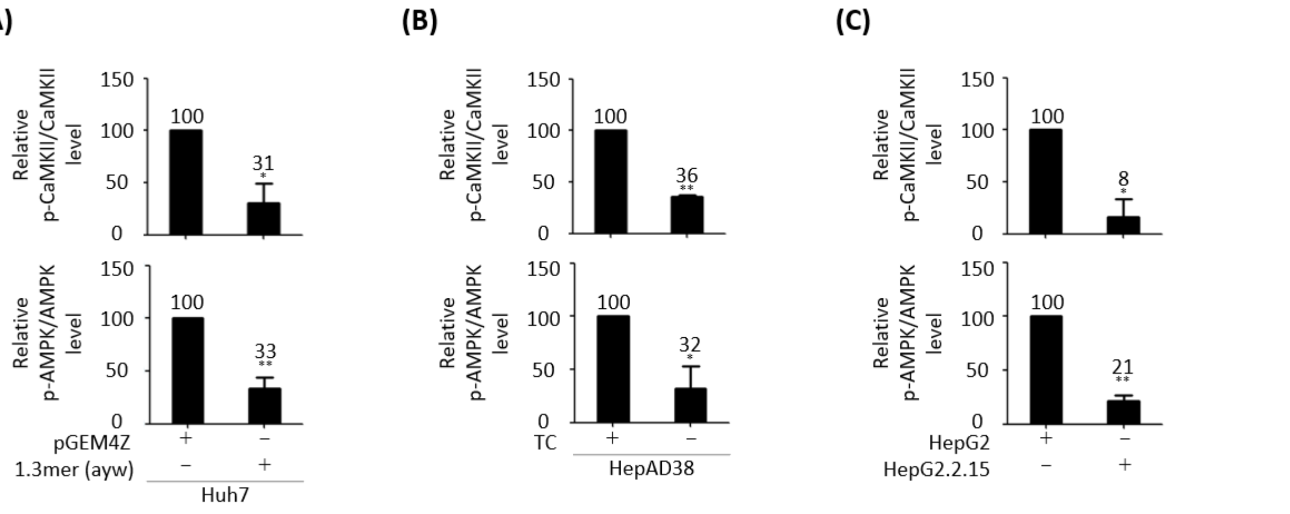

Figure S1. Relative levels of total and active CaMKII and AMPK in HBV replicating cells presented in Figure 3. Phosphorylated level was normalized to total level from panels 1–2 and 3–4 in Figure 3. * $p < 0.05$, ** $p < 0.005$ relative to respective control by Student's t-tests ($n = 3$). The bars represent means ± SD of three independent experiments.

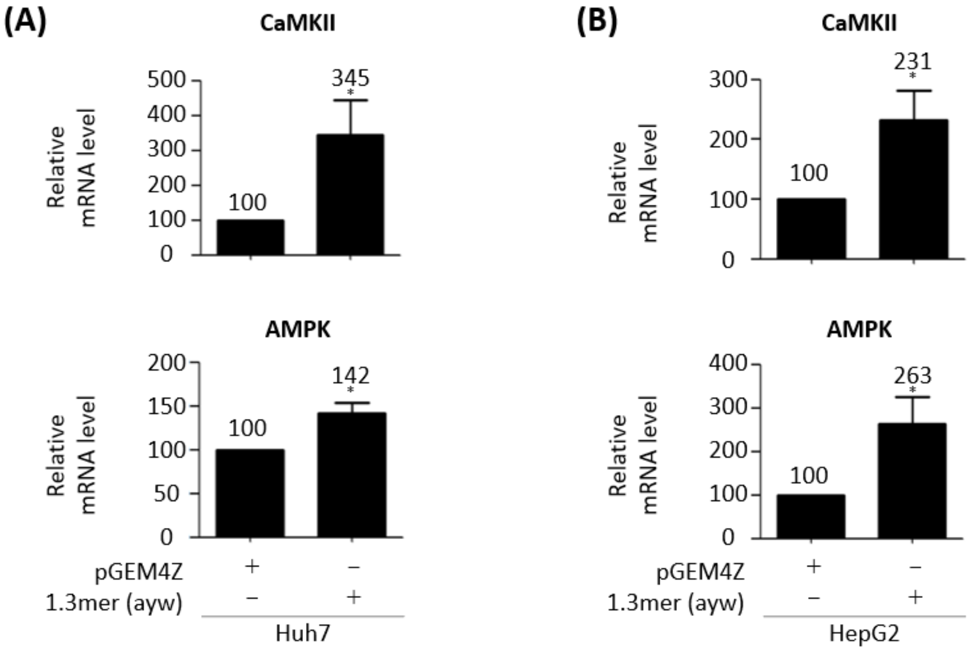

Figure S2. Quantitative real-time RTPCR results to show the levels of CaMKII and AMPK mRNAs in HBV replicating cells. CaMKII and AMPK mRNA levels were increased in HBV replicating Huh7 cells (A) and HepG2 cells (B). The relative mRNA levels were quantified by normalization to actin (loading control). * $p < 0.05$ relative to respective control by Student's t-tests ($n = 3$). The bars represent means ± SD of three independent experiments.

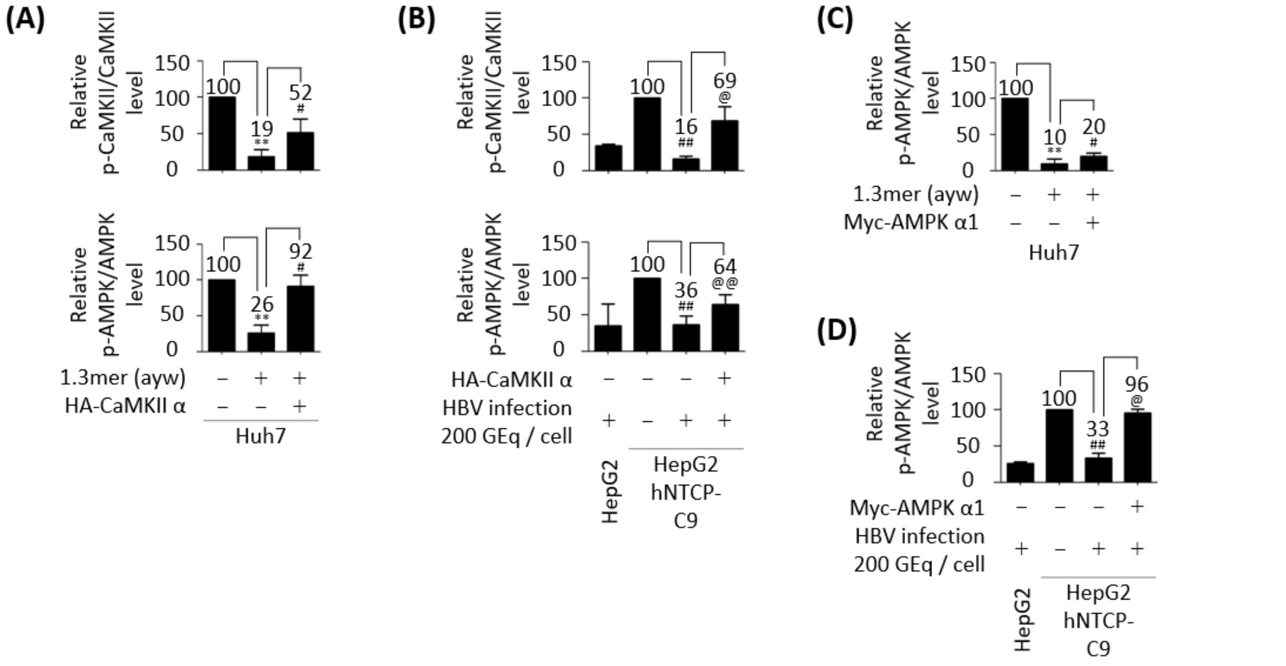

Figure S3. Relative levels of total and active CaMKII and AMPK in HBV replicating cells presented in Figures 4C–D and 5C–D. (A) Huh7 cells were (co)-transfected as in Figure 4C. (B) HepG2 and HepG2-hNTCP-C9 cells were infected as in Figure 4D. (C) Huh7 cells were (co)-transfected as in Figure 5C. (D) HepG2 and HepG2-hNTCP-C9 cells were infected as in Figure 5D. Phosphorylated level was normalized to total level. $^{**}p < 0.005$ relative to mock–transfected control by Student's t-tests (lane 1 *vs*. 2). $^{\#}p < 0.05$, $^{\#\#}p < 0.005$ relative to corresponding control by Student's t-tests (lane 2 *vs*. 3). $^{@}p < 0.05$, $^{@@}p < 0.005$ relative to corresponding control by Student's t-tests (lane 3 *vs*. 4) ($n = 3$). The bars represent means ± SD of three independent experiments.

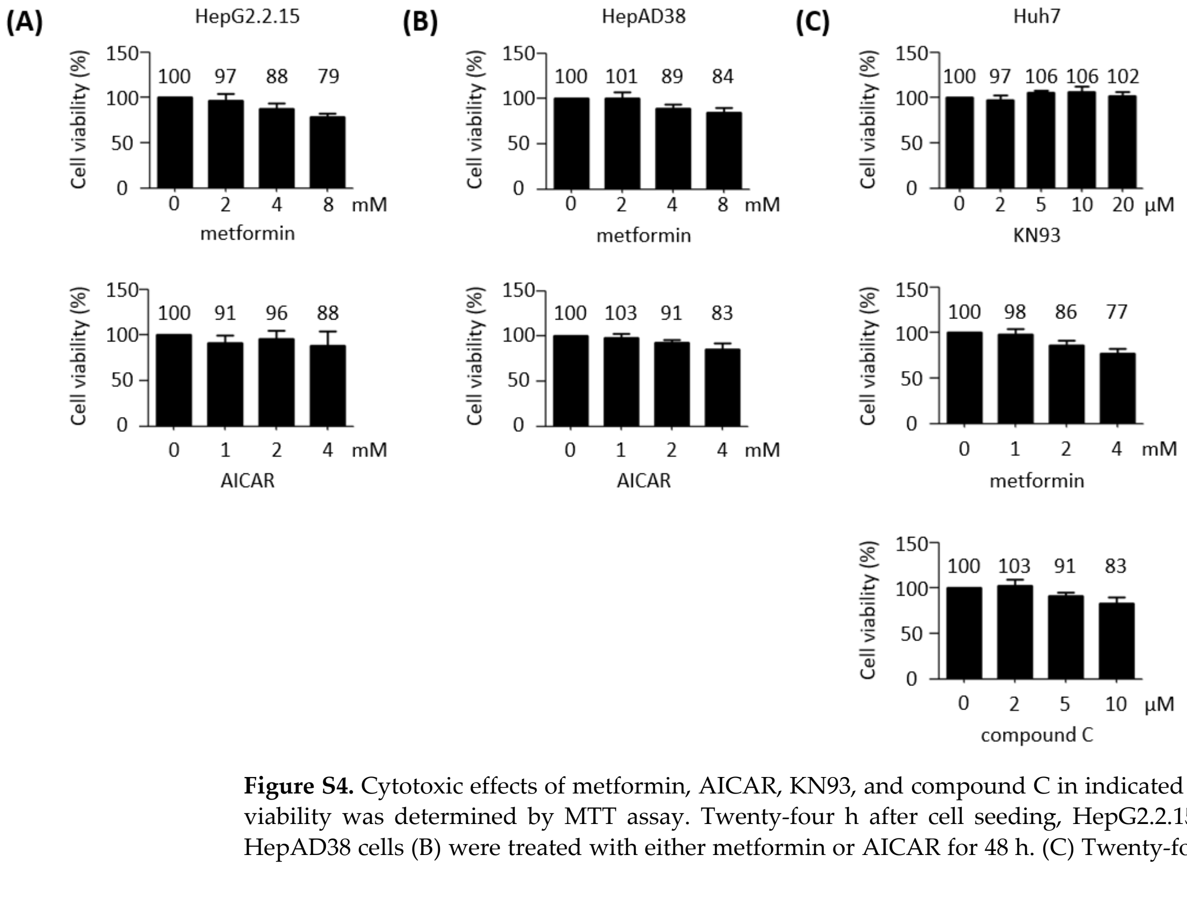

Figure S4. Cytotoxic effects of metformin, AICAR, KN93, and compound C in indicated cells. Cell viability was determined by MTT assay. Twenty-four h after cell seeding, HepG2.2.15 (A) and HepAD38 cells (B) were treated with either metformin or AICAR for 48 h. (C) Twenty-four h after

cell seeding, Huh7 cells were (co-)transfected. Twenty-four h post-transfection, Huh7 cells were treated with KN93, metformin, and compound C for 48 h. The bars represent means ± SD of three independent experiments.

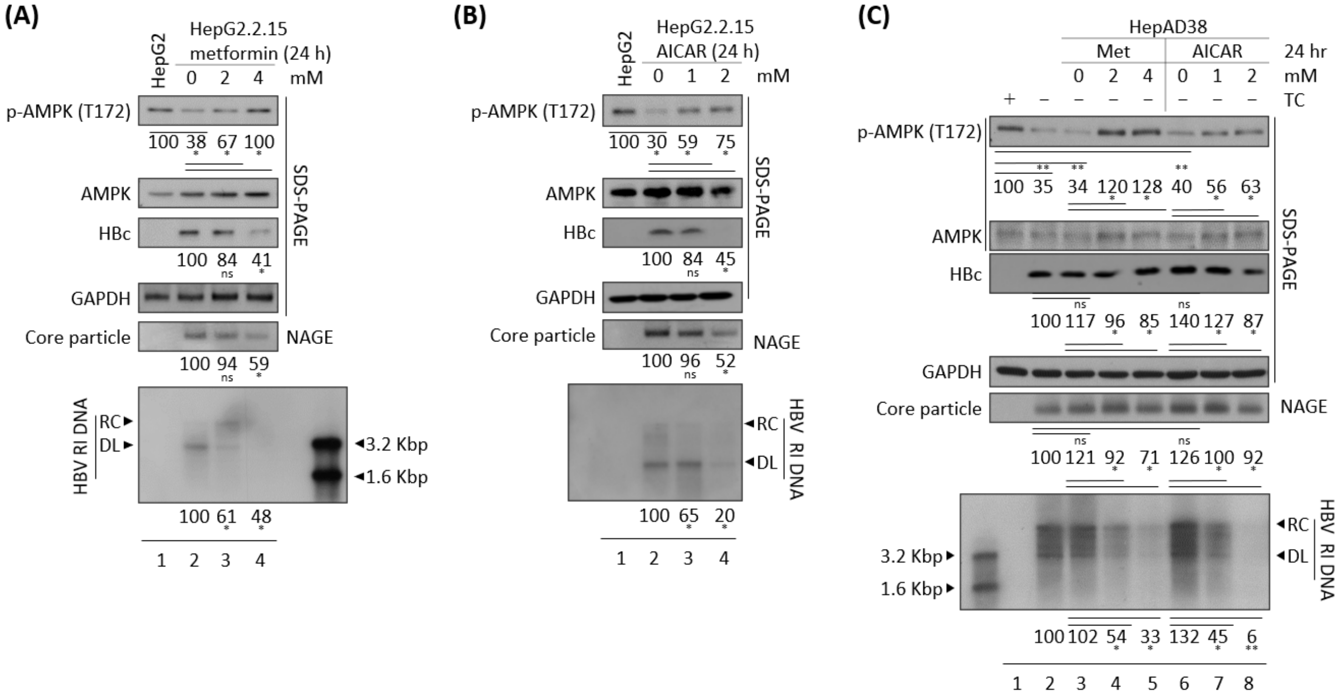

Figure S5. Activation of AMPK reduces HBV replication. (A) Metformin, an AMPK activator, inhibits HBV replication in HepG2.2.15 cells. (B) AICAR, an AMPK activator, inhibits HBV replication in HepG2.2.15 cells. (C) Metformin or AICAR inhibits HBV replication in HepAD38 cells. HepG2 (lanes 1), a negative control, and HepG2.2.15 (lanes 2–4) cells were incubated for 48 h (A and B). HepAD38 cells were incubated with (lane 1) or without TC (lanes 2–8) for 48 h (C). HepG2.2.15 and HepAD38 cells were treated with 0, 2, or 4 mM metformin (A and C), or 0, 1, or 2 mM AICAR (B and C) for 24 h. NAGE and immunoblotting for core particles and Southern blotting for HBV DNA synthesis were performed as described in Figure 1. Indicated proteins were detected by Western blotting using primary antibodies. Relative expression was quantified by normalization to GAPDH (loading control) using ImageJ 1.50b software. ns, not significant; * $p < 0.05$, ** $p < 0.005$ relative to respective control by Student's t-tests ($n = 3$).

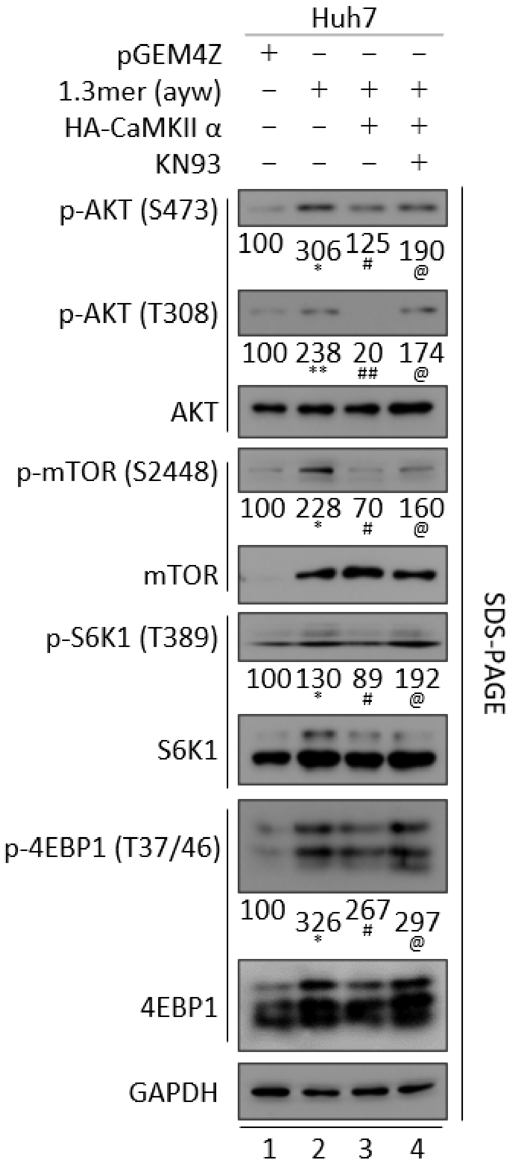

Figure S6. AKT-mTOR/S6K1/4EBP1 signaling pathway presented in Figure 6. $^{*}p < 0.05$, $^{**}p < 0.005$ relative to mock-transfected control by Student's t-tests (lane 1 *vs.* 2). $^{\#}p < 0.05$, $^{\#\#}p < 0.005$ relative to corresponding control by Student's t-tests (lane 2 *vs.* 3). $^{@}p < 0.05$ relative to corresponding control by Student's t-tests (lane 3 *vs.* 4) ($n = 3$).

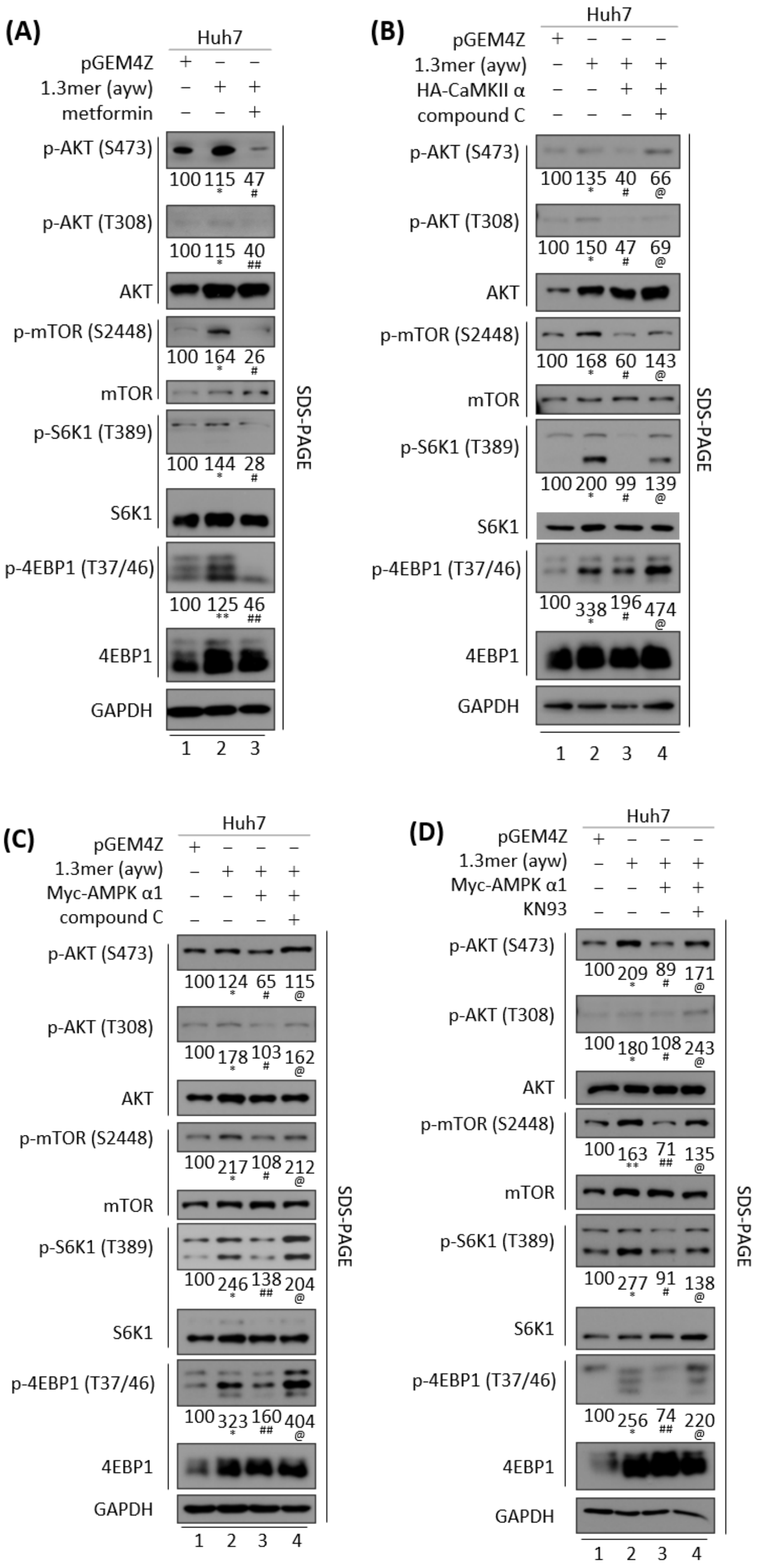

Figure S7. AKT-mTOR/S6K1/4EBP1 signaling pathway presented in Figures 7 (A and B) and 8 (C and D). $^{*}p < 0.05$, $^{**}p < 0.005$ relative to mock-transfected control by Student's t-tests (lane 1 *vs*. 2). $^{\#}p < 0.05$, $^{\#\#}p < 0.005$ relative to corresponding control by Student's t-tests (lane 2 *vs*. 3). $^{@}p < 0.05$ relative to corresponding control by Student's t-tests (lane 3 *vs*. 4) ($n = 3$).

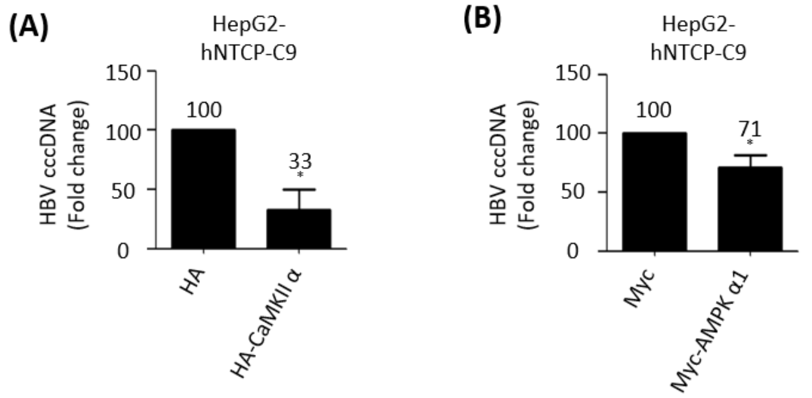

Figure S8. Quantitative real-time PCR of HBV cccDNA in HBV-infected cells from Figures 9C and 10C. Overexpression of CaMKII (A) or AMPK (B) decreased HBV cccDNA levels. HBV cccDNA was quantified by normalization to actin (loading control). * $p < 0.05$ relative to respective control by Student's t-tests ($n = 3$). The bars represent means ± SD of three independent experiments.

Table S1. Primers for construction of CaMKII α and AMPK α1 expression plasmids, qPCR, and RT-qPCR.

Constructs	Sequence (5′→3′)
HA–CaMKII α	
Forward	CCG AAT TCT TAT GGC TAC AA
Reverse	CCG AAT TCT CAA TGG GGC AG
pCDH–HA–CaMKII α	
Forward	ATT CTA GAA TGT ACC CAT AC
Reverse	GCG GAT CCT CAA TGG GGC AG
Myc–AMPK α1	
Forward	ATG CTG GCC ATG GCG ACA GCC GAG A
Reverse	ATG CGA ATT CTT ATT GTG CAA GAA T
pCDH–Myc–AMPK α1	
Forward	ATG CTC TAG AAT GGC ATC AAT GCA G
Reverse	ATG CGA ATT CTT ATT GTG CAA GAA T
cccDNA	
Forward	CTCCCCGTCTGTGCCTTCT
Reverse	GCCCCAAAGCCACCCAAG
CaMKII α	
Forward	CCT GTA CAT CCT GCT GGT TGG G
Reverse	TTG ATC AGA TCC TTG GCT TCC
AMPK α1	
Forward	CTCTATGCTTTATTATGTGG
Reverse	GATCCTGGTGATTTCTGTTG
Actin	
Forward	CATGTACGTTGCTATCCAGGC
Reverse	CTCCTTAATGTCACGCACGAT

Table S2. Antibodies used for this study.

Antibody target	Species	Exp	Supplier	Catalog no. or reference
HBc	Rabbit polyclonal	SDS-PAGE-IB	In-house	[40]
GAPDH	Mouse monoclonal	SDS-PAGE-IB	Santa Cruz	sc32233
HA	Mouse monoclonal	SDS-PAGE-IB	Abcam	ab18181
Myc	Mouse monoclonal	SDS-PAGE-IB	Santa Cruz	sc40
p-CaMKII (T286)	Rabbit polyclonal	SDS-PAGE-IB	Cell Signaling Technology	#3361
CaMKII	Rabbit polyclonal	SDS-PAGE-IB	Cell Signaling Technology	#3362
p-AMPK α (T172)	Rabbit polyclonal	SDS-PAGE-IB	Cell Signaling Technology	#2535
AMPK α	Rabbit polyclonal	SDS-PAGE-IB	Cell Signaling Technology	#2532
p-AKT (S437)	Rabbit monoclonal	SDS-PAGE-IB	Cell Signaling Technology	#9271
p-AKT (T308)	Rabbit polyclonal	SDS-PAGE-IB	Cell Signaling Technology	#9275
AKT	Rabbit polyclonal	SDS-PAGE-IB	Cell Signaling Technology	#9272
p-mTOR (S2448)	Rabbit polyclonal	SDS-PAGE-IB	Cell Signaling Technology	#2971
mTOR	Rabbit polyclonal	SDS-PAGE-IB	Cell Signaling Technology	#2972
p-S6K1 (T389)	Rabbit polyclonal	SDS-PAGE-IB	Cell Signaling Technology	#9205
S6K1	Rabbit polyclonal	SDS-PAGE-IB	Cell Signaling Technology	#9202
p-4EBP1 (T37/46)	Rabbit polyclonal	SDS-PAGE-IB	Cell Signaling Technology	#9459
4EBP1	Rabbit polyclonal	SDS-PAGE-IB	Cell Signaling Technology	#9452

Table S3. Clinical characteristics of HBV-associated HCC patients presented in Figure 2.

Patient No.	Age	Sex	ALT	AST	HBV DNA (IU/ml)
15	62	M	29	64	6,400,000
19	50	F	40	28	287,000
25	48	M	30	102	939,000
6	35	M	57	36	3990
10	51	M	33	26	0
13	52	M	29	34	48
14	55	M	155	222	0
20	58	F	47	46	85,800
21	47	M	54	57	287
26	58	M	35	62	41,200
27	65	M	22	24	0
34	45	M	70	34	0

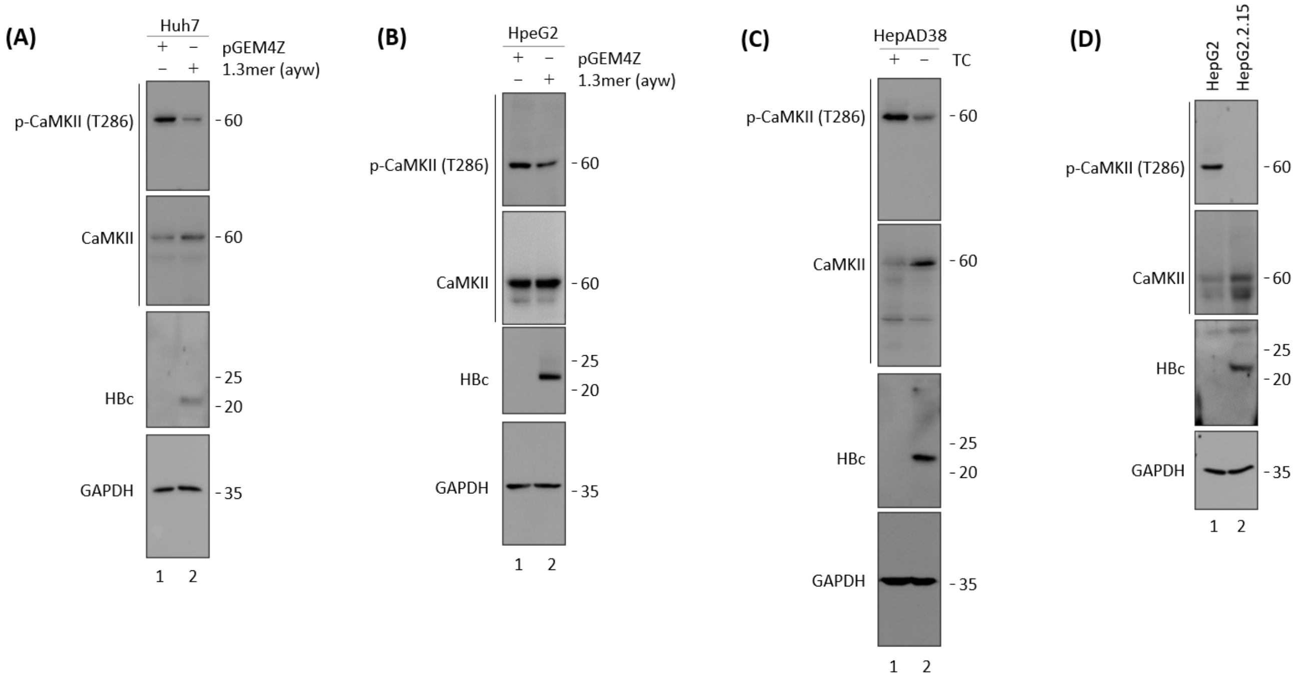

Figure S9. Uncut scans of original western blotting images of Figure 1.

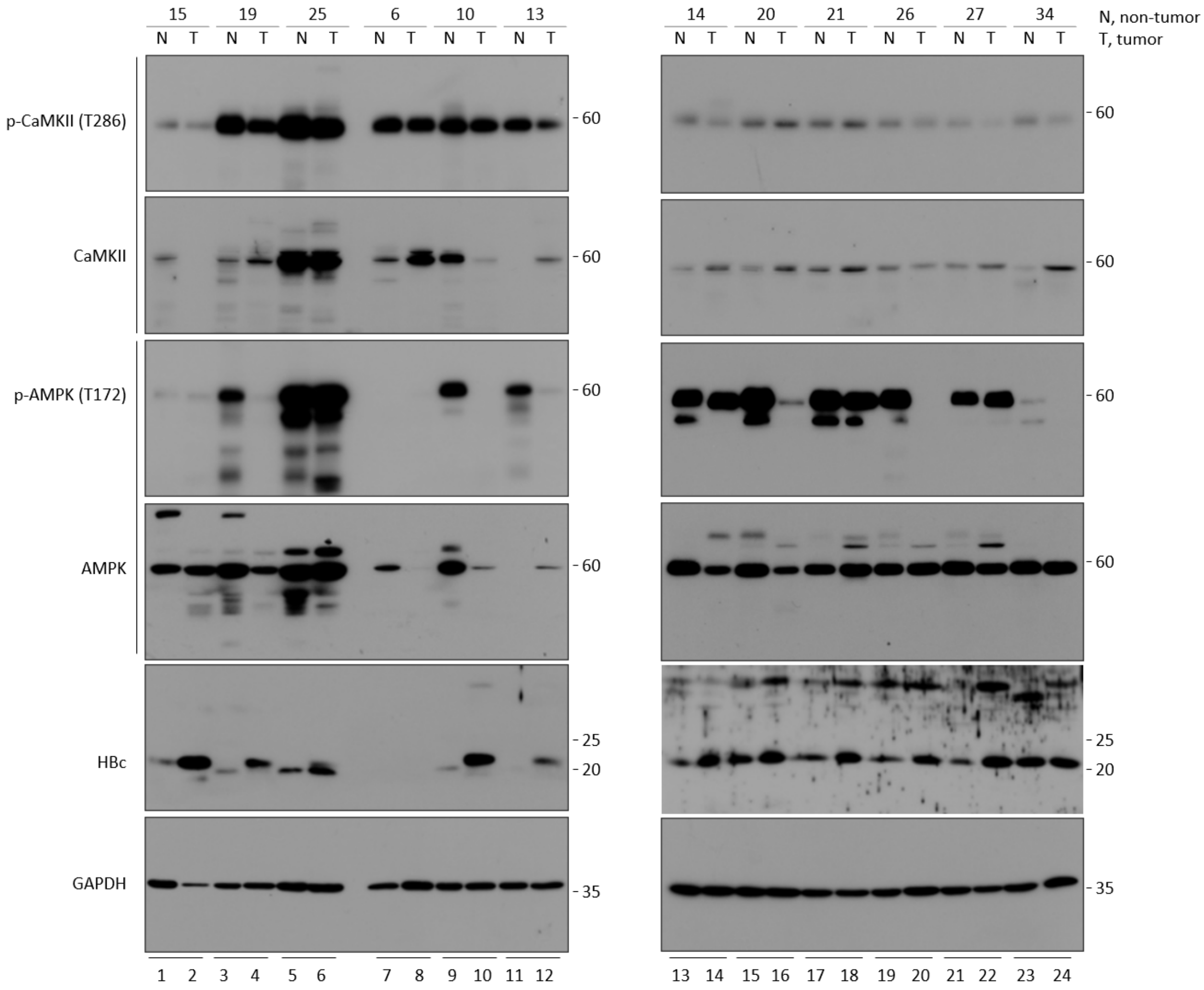

Figure S10. Uncut scans of original western blotting images of Figure 2.

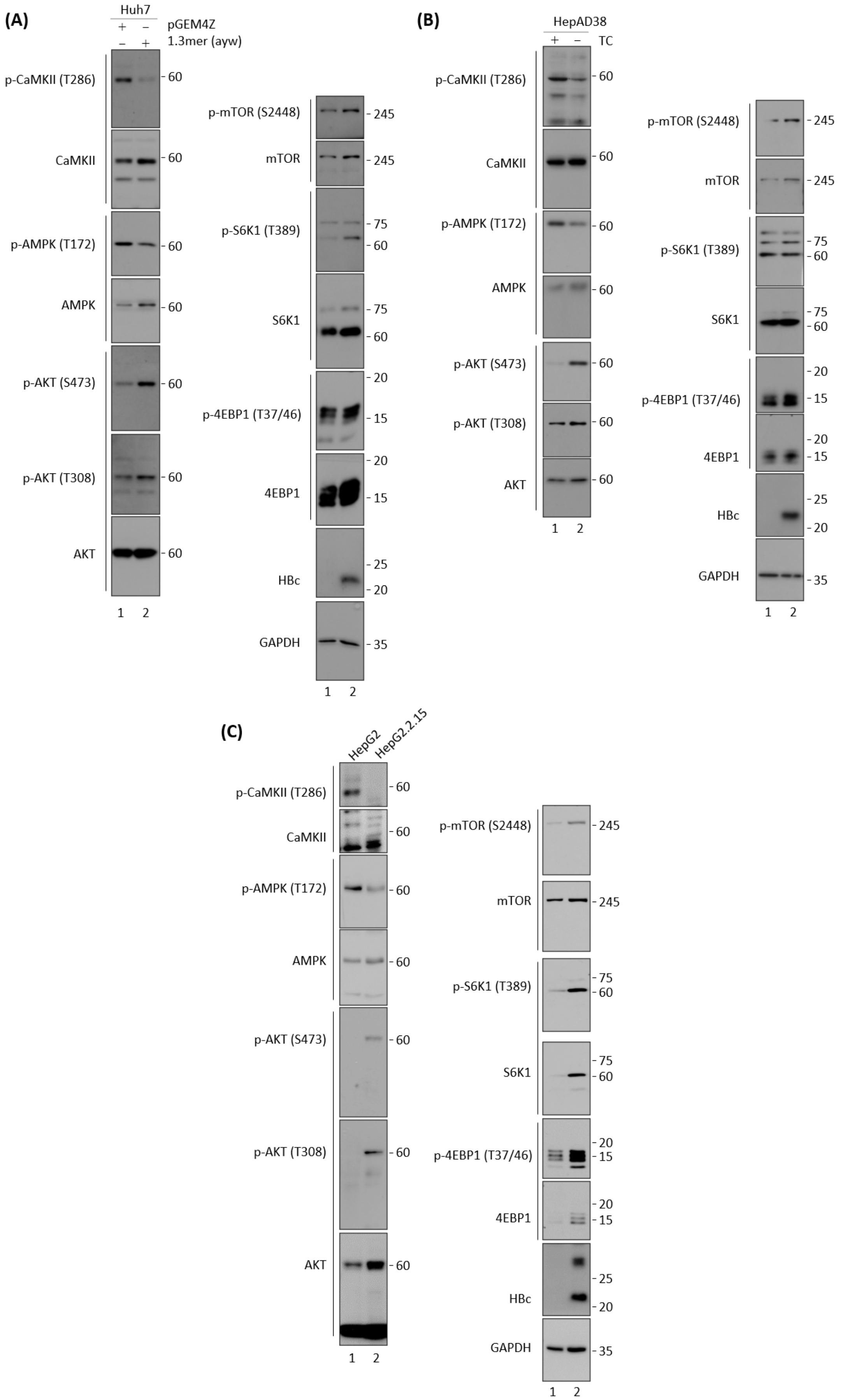

Figure S11. Uncut scans of original western blotting images of Figure 3.

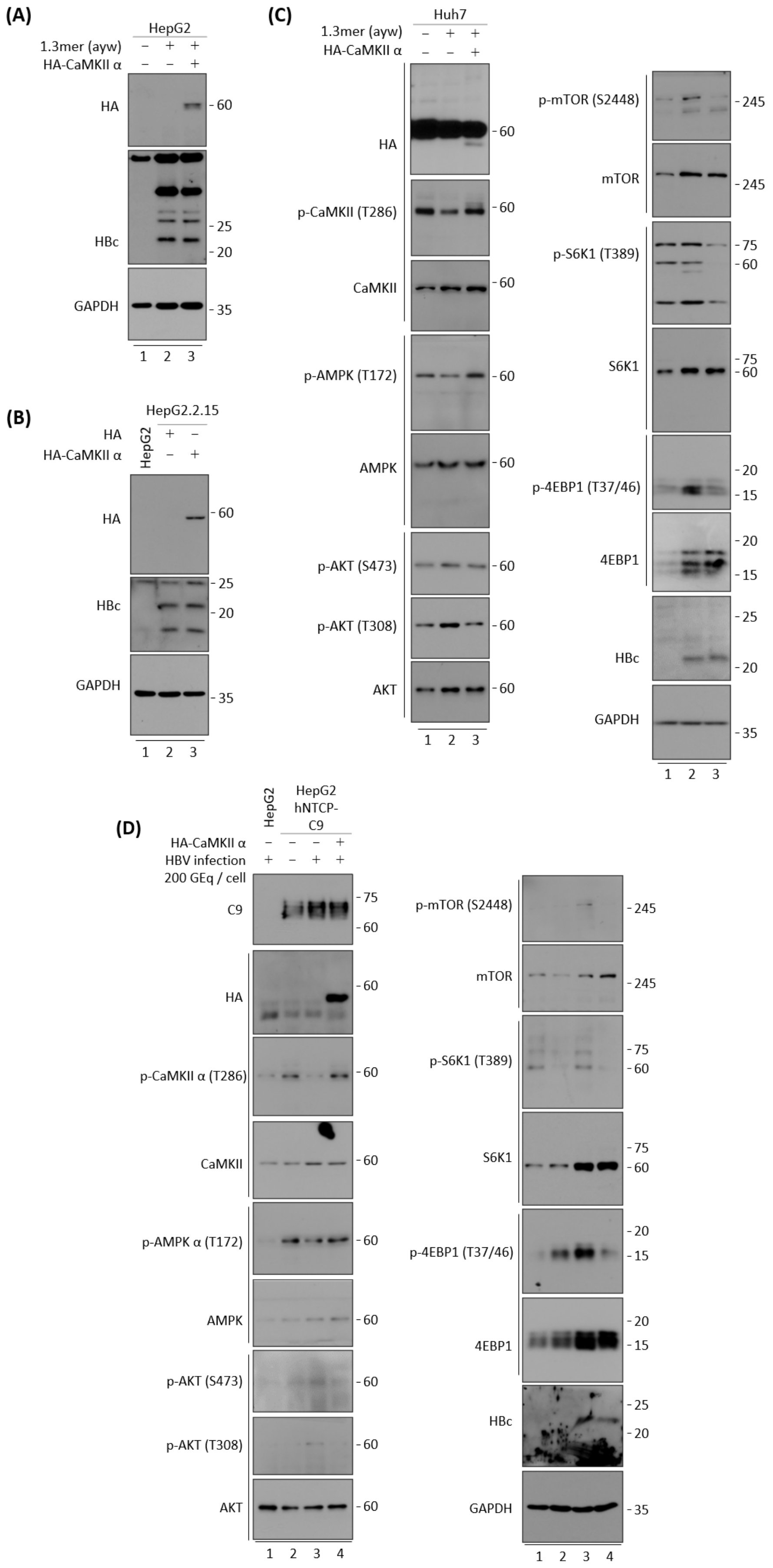

Figure S12. Uncut scans of original western blotting images of Figure 4.

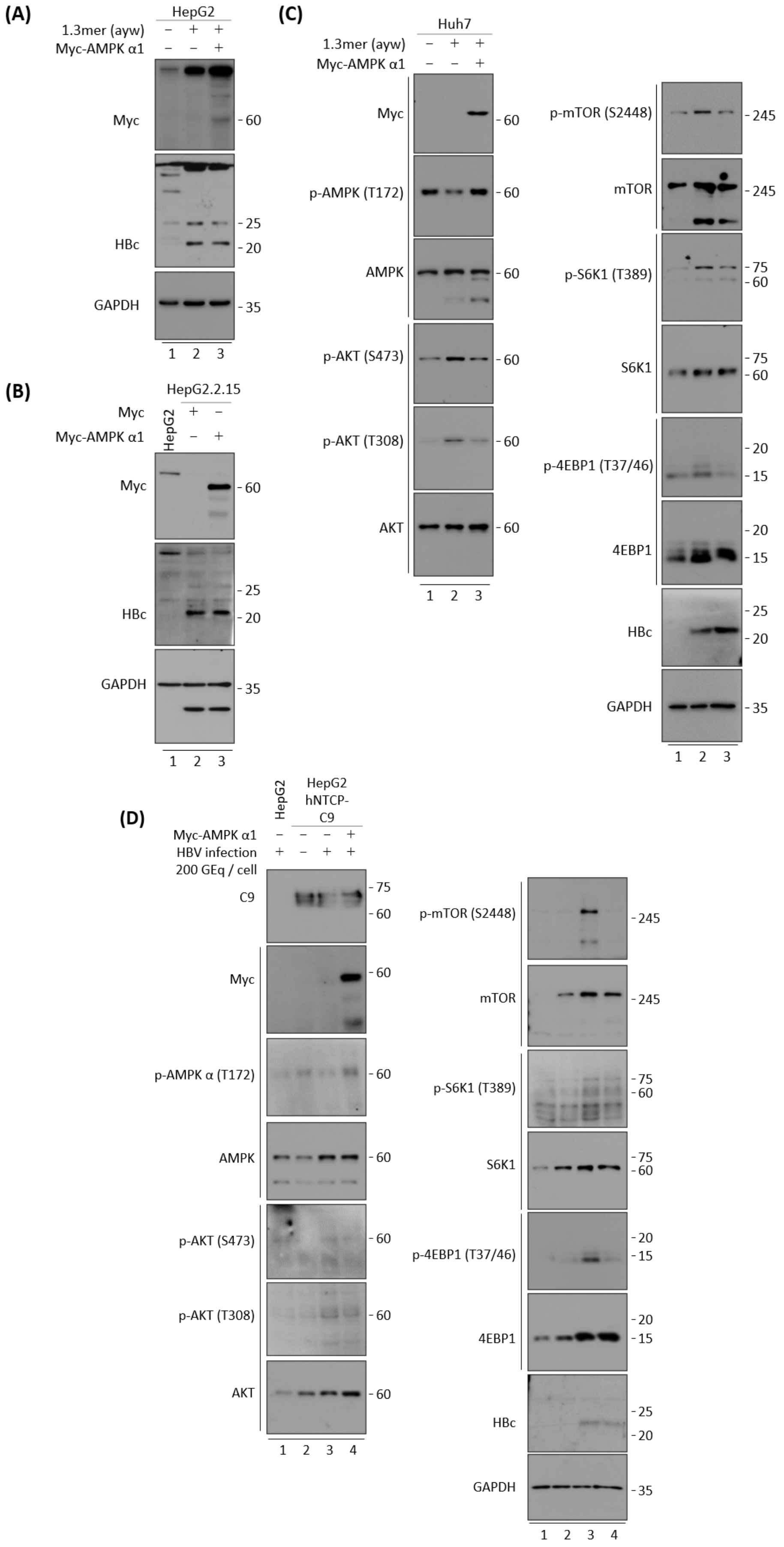

Figure S13. Uncut scans of original western blotting images of Figure 5.

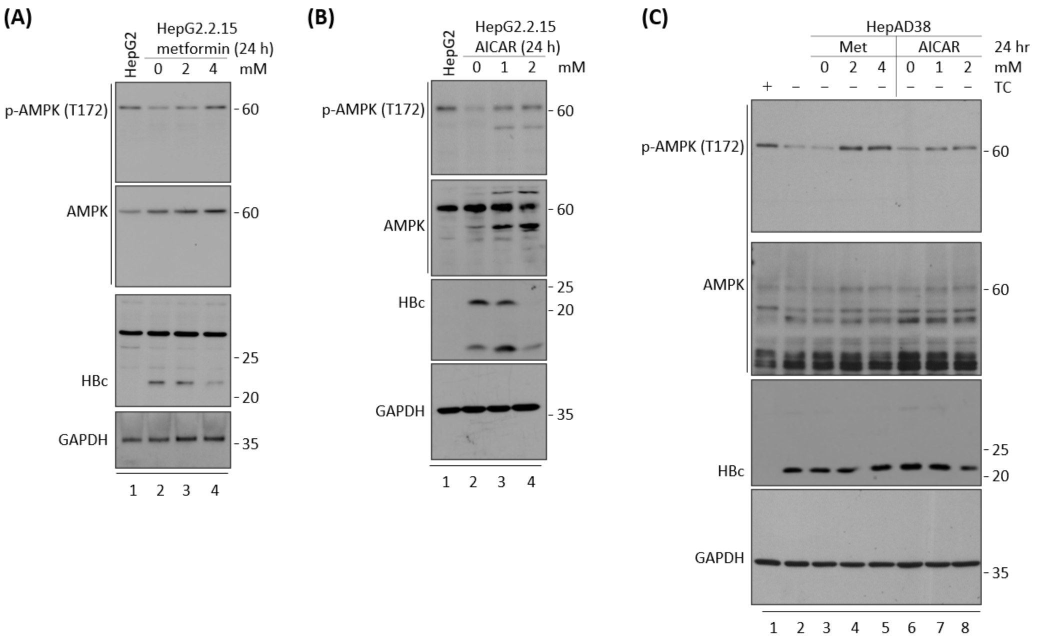

Figure S14. Uncut scans of original western blotting images of Figure S5.

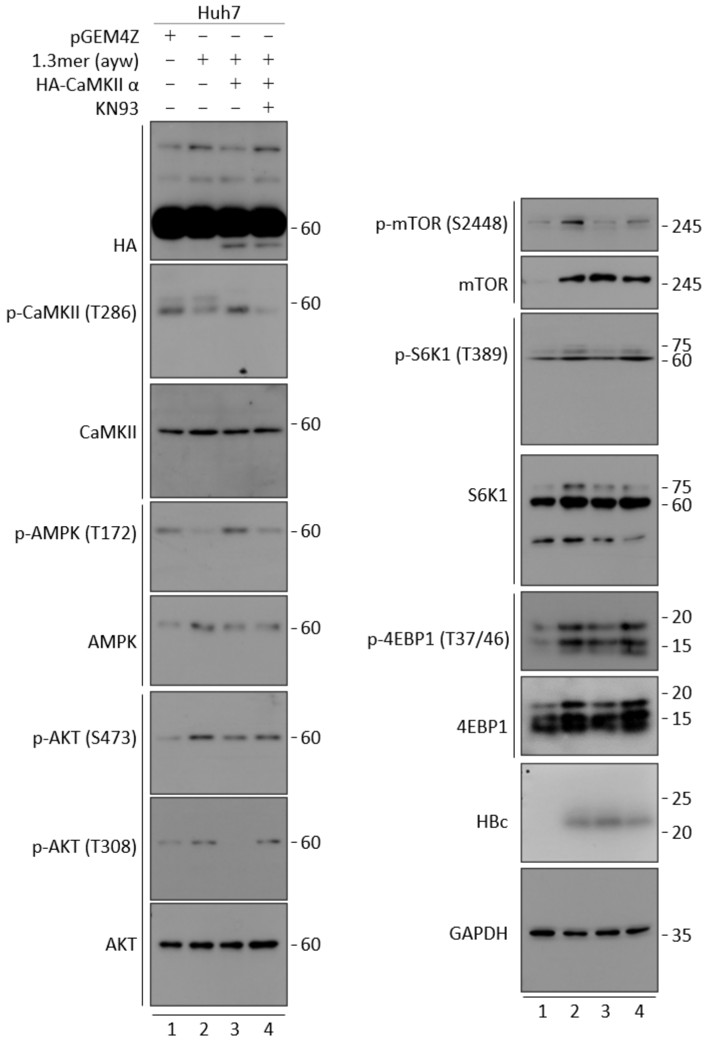

Figure S15. Uncut scans of original western blotting images of Figure 6 and S6.

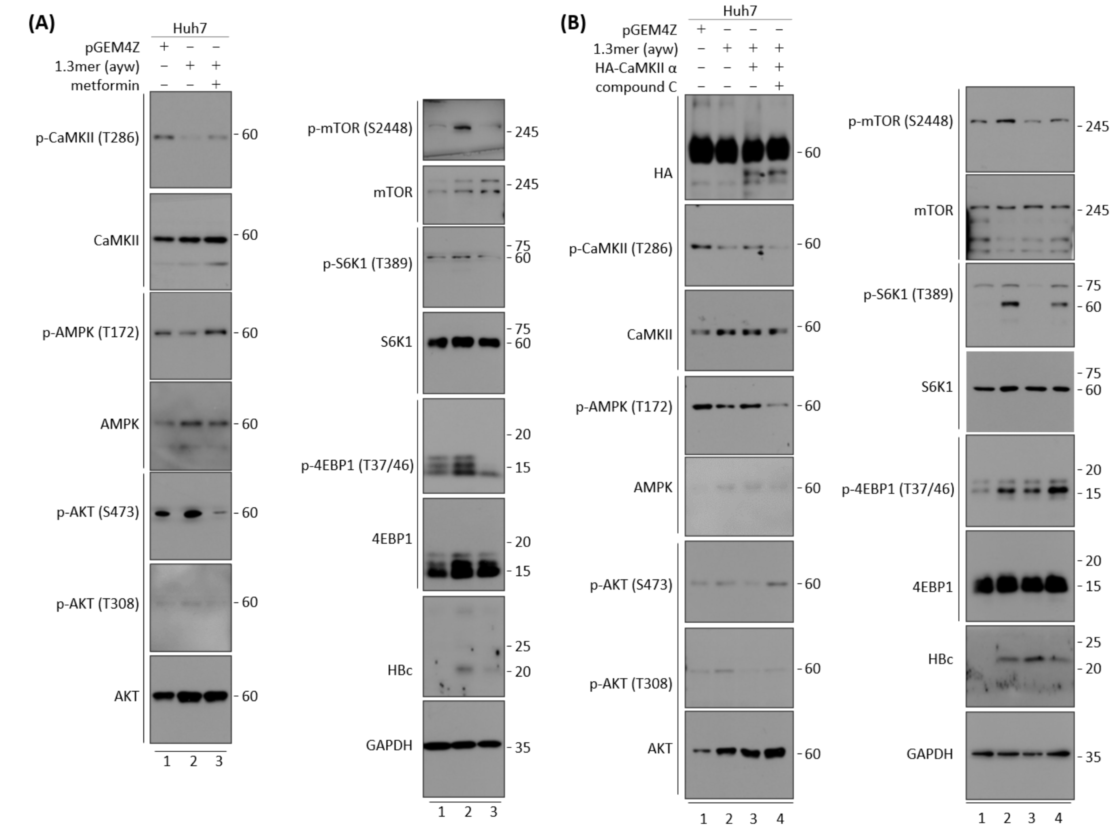

Figure S16. Uncut scans of original western blotting images of Figure 7, S7A, and S7B.

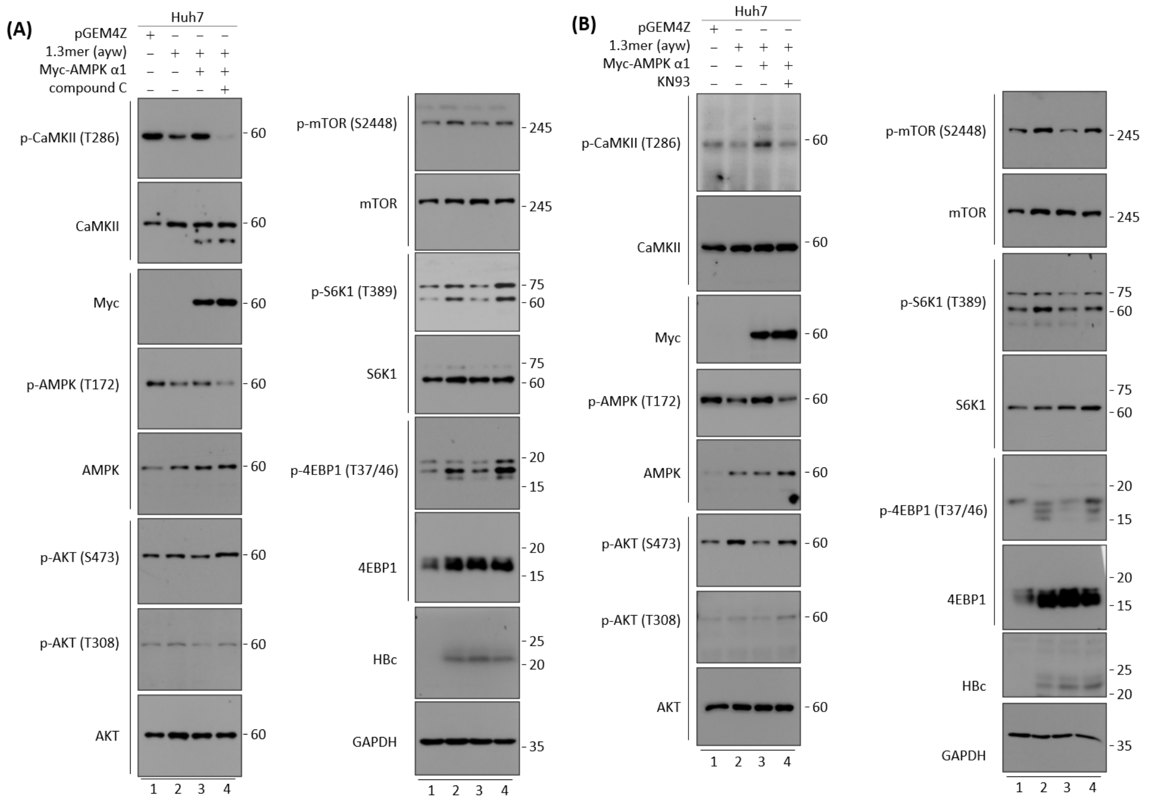

Figure S17. Uncut scans of original western blotting images of Figure 8, S7C, and S7D.

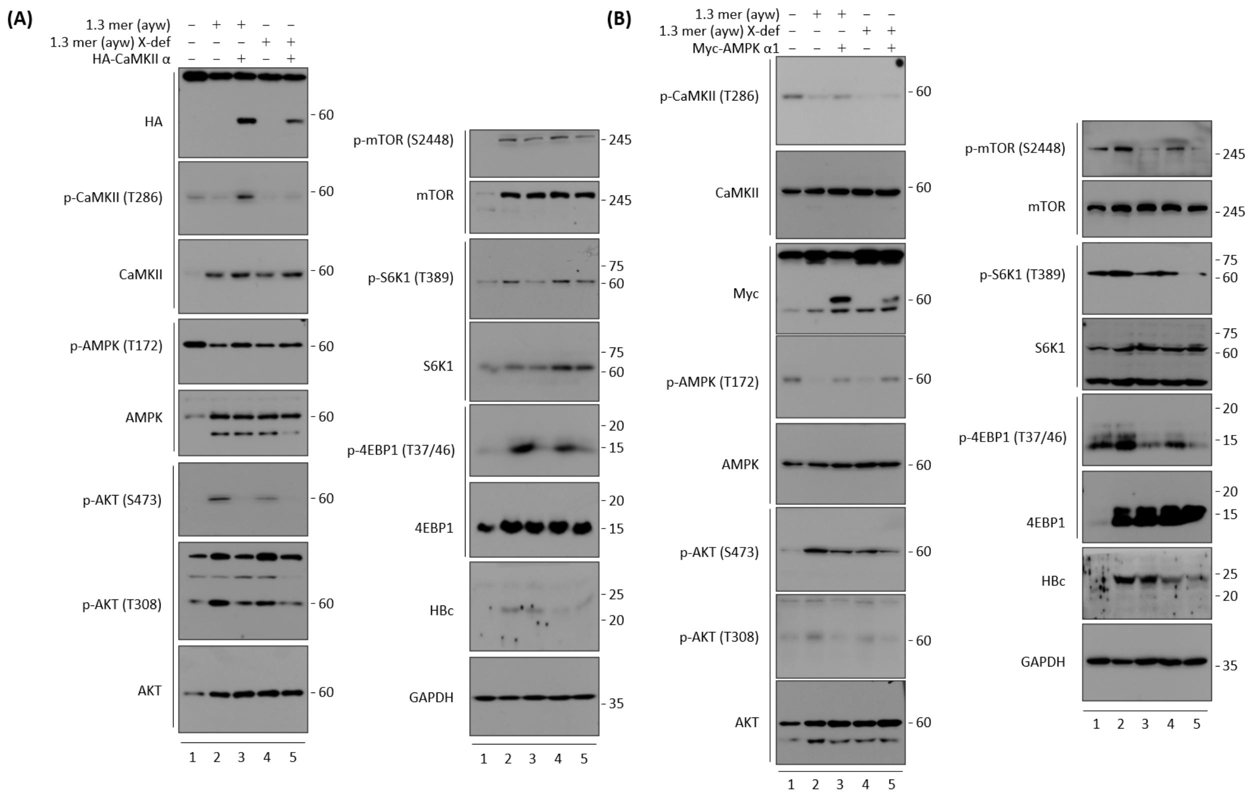

Figure S18. Uncut scans of original western blotting images of Figure 11.
